# Supplementary material for: Concerted oxygen diffusion across heterogeneous oxide interfaces for intensified propane dehydrogenation
Source: Nat Commun. 2023 May 5;14:2620. doi: 10.1038/s41467-023-38284-0 (PMC10163216; doi:10.1038/s41467-023-38284-0)
Supplement: Supplementary file 1 — Supplementary Information [file 41467_2023_38284_MOESM1_ESM.pdf]

Supporting Information to

Concerted Oxygen Diffusion across Heterogeneous Oxide Interfaces for Intensified Propane Dehydrogenation

Sai Chen[1,2,3], Ran Luo[1,2,3], Zhi-Jian Zhao[1,2], Chunlei Pei[1,2], Yiyi Xu[1,2], Zhenpu Lu[1,2], Chengjie Zhao[1,2], Hongbo Song[1,2], and Jinlong Gong[1,2,3*]

[1]*Key Laboratory for Green Chemical Technology of Ministry of Education, School of Chemical Engineering & Technology, Collaborative Innovation Center for Chemical Science & Engineering, Tianjin University, Tianjin 300072, China*

[2]*Collaborative Innovation Center for Chemical Science & Engineering (Tianjin), Tianjin 300072, China*

[3]*Joint School of National University of Singapore and Tianjin University, International Campus of Tianjin University, Binhai New City, Fuzhou 350207, China*

*Correspondence: jlgong@tju.edu.cn

Figure S1 | Textural properties of ceria-vanadia redox catalysts........................................3
Figure S2 | Electronic property of ceria-vanadia redox catalysts. 5
Figure S3 Schematic cyclogram, effects of V contents, Ce contents, WHSV of propane 6
Figure S4 | Reaction performance during the CL-ODH processes. 8
Figure S5 | Propane conversion and propylene selectivity and the deactivation rates. ...9
Figure S6 | Propylene formation rates as a function of deactivation rate. 10
Figure S7 | Structure evolution during the dehydrogenation and redox cycles. 11
Figure S8 | The propylene formation over heterogeneous oxygen carrier composites. .12
Figure S9 | Reaction performance of ethane on ceria-vanadia redox catalysts. 13
Figure S10 | ASPEN Plus simulation. 14
Figure S11 | Experimental evidence of oxygen transfer behavior. 15
Figure S12 | *in situ* Raman spectra during propane dehydrogenation. 16
Figure S13 | The formation of H_2O and H_2, and C_3H_6 formation rates. 17
Figure S14 | Temperature-programmed surface reaction and transient pulses. 18
Figure S15 | NH_3-TPD profiles 19
Figure S16 | Oxygen release kinetics. 20
Figure S17 | The determined consumption amount and rates of oxygen species. 21
Figure S18 | The Arrhenius plots of k_{chem} and D_{diff} from 550 to 600 °C. 22
Figure S19 | Reduction process and relative oxygen formation energies. 23
Figure S20 | Bader charge analysis. 24
Figure S21 | O_{vac} formation energies of bulk V_2O_5, VO_2, V_2O_3, and CeO_2.......................25
Figure S22 | Oxygen diffusion pathway derived by DFT calculations. 26
Figure S23 | Unit [VO_4] rings in ML-VO_2....27
Figure S24 | Reaction energy profiles of H_2O formation and H_2 formation 28
Figure S25 | Calculated potential energy diagram. 29
Table S1. Physicochemical properties of vanadia-ceria redox catalysts...........................30
Table S2. XPS quantification....31
Table S3. Comparison of VO_x-CeO_2 catalysts with state-of-the-art catalysts..................32
Table S4. Representative oxide-based catalysts used in propane dehydrogenation.........34
Table S5. BET and pore size changes of vanadia-ceria catalysts during redox cycles....39
Table S6. Simulation Settings (modules and methods) by ASPEN plus...........................40
Table S7. Simulation conditions and assumptions by ASPEN plus.................................41
Table S8. Simulation Settings (feeds and yields)...42
Table S9. Comparison of energy consumption of non-oxidative PDH and CL-ODH.....43
Table S10. Comparison of energy consumption of non-oxidative PDH and CL-ODH...44
Table S11. The surface reaction coefficient (k_{chem}) and bulk diffusion coefficient (D_{diff}).45
Table S12. Bader charge analysis 46
Supplementary References...47

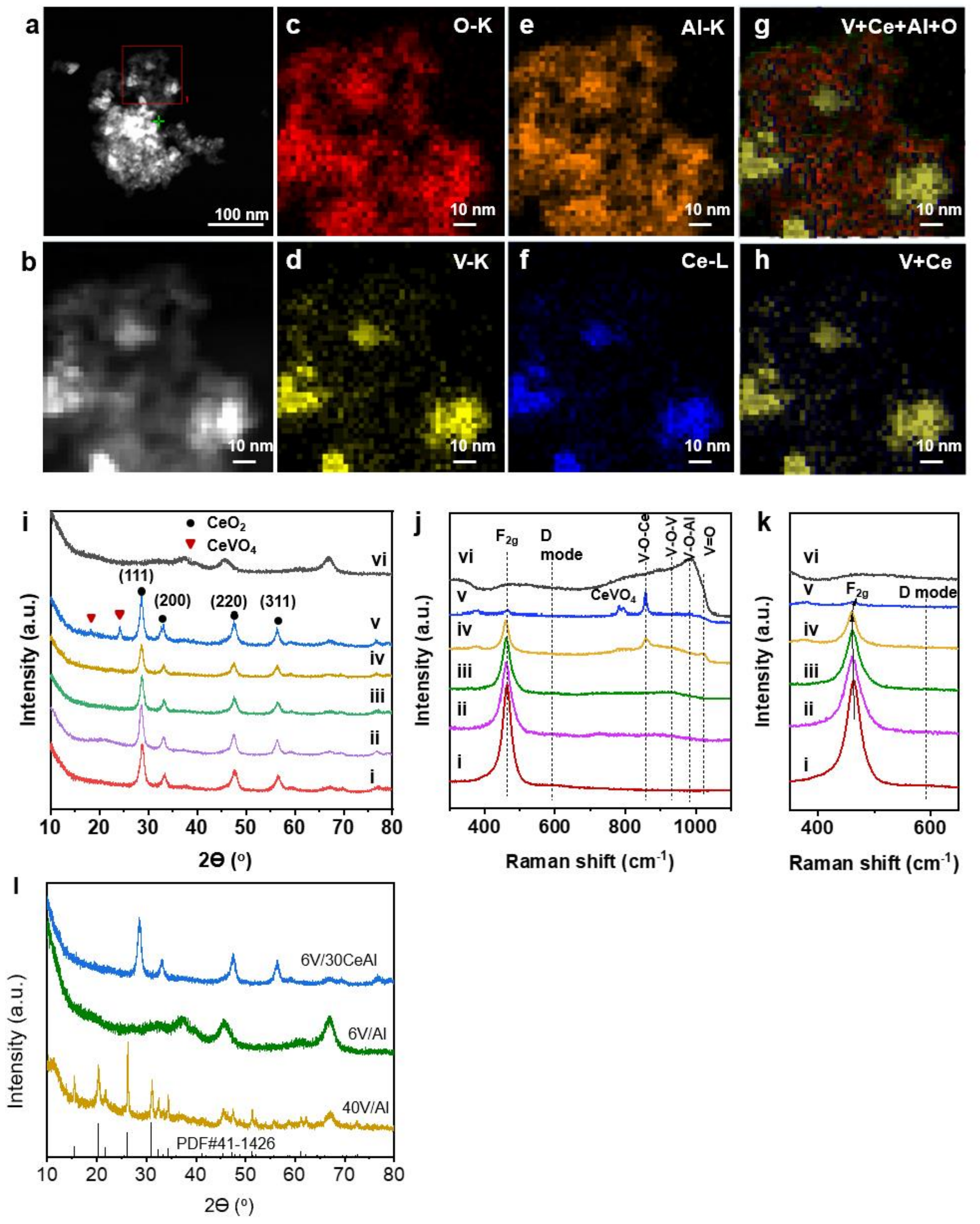

Figure S1 | Textural properties of ceria-vanadia redox catalysts. (a, b) TEM images and (c-f) EDS mappings of ceria-vanadia redox catalysts (6V/30CeAl) (e, O; f, V; g, Al; h: Ce). (i) (g) Overlap of V, Ce, Al, and O; (h) Overlap of V and Ce. XRD patterns, (j) Raman spectra, and (k) the enlarged Raman spectra of vanadia (6V/Al0, ceria (30CeAl), and ceria-vanadia redox catalysts supported on Al_2O_3 with increasing V loadings (i-vii, 30CeAl, 0.5V/30CeAl, 3V/30CeAl, 6V/30CeAl, 12V/30CeAl and 6V/Al). (l) XRD patterns of 6V/Al, 40V/Al and 6V/30CeAl.

EDS mappings showed that the element of Ce and V are well distributed and overlapped among the different particles, indicating VO_x were highly dispersed on the

surface of CeO_2 to form the core-shell structures, which persists in every VO_x/CeO_2 particle. XRD patterns showed that neither crystalline V_2O_5 nor $CeVO_4$ was observed when the percent weight ratio of V/Al_2O_3 was higher than 6. Raman spectra showed that in addition to CeO_2 features, it appears new Raman bands in the range of 800-1,200 cm^{-1}: V=O stretching (960-1,050 cm^{-1}) and bridging modes, such as V-O-V (920 cm^{-1}) and V-O-Ce (859 cm^{-1}). The shift of F_{2g} band in CeO_2 from 464 to 459 cm^{-1} implies that VO_x interacts with CeO_2 to deform the fluorite-like structure. We note that for VO_x supported on Al_2O_3, the band of V-O-Al is dominated. However, with CeO_2 addition, the band of V-O-Al disappeared, and V-O-Ce emerged, which suggests that VO_x well interact with CeO_2 but not Al_2O_3, corresponding with the TEM mappings where V and Ce are well overlapped.

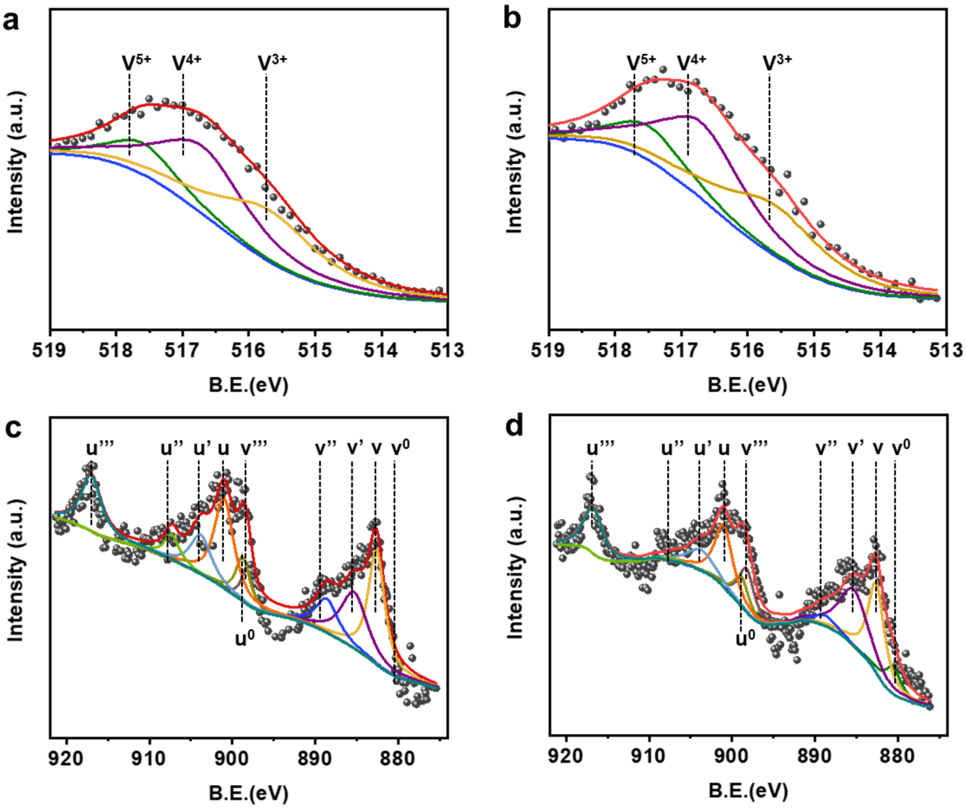

Figure S2 | Electronic property of ceria-vanadia redox catalysts. (a) and (b) XPS spectra of V *2p* over vanadia (6V/Al) and ceria-vanadia redox catalysts (6V/30CeAl). (c) and (d) XPS spectra of Ce *3d* over ceria (30CeAl) and ceria-vanadia redox catalysts (6V/30CeAl).

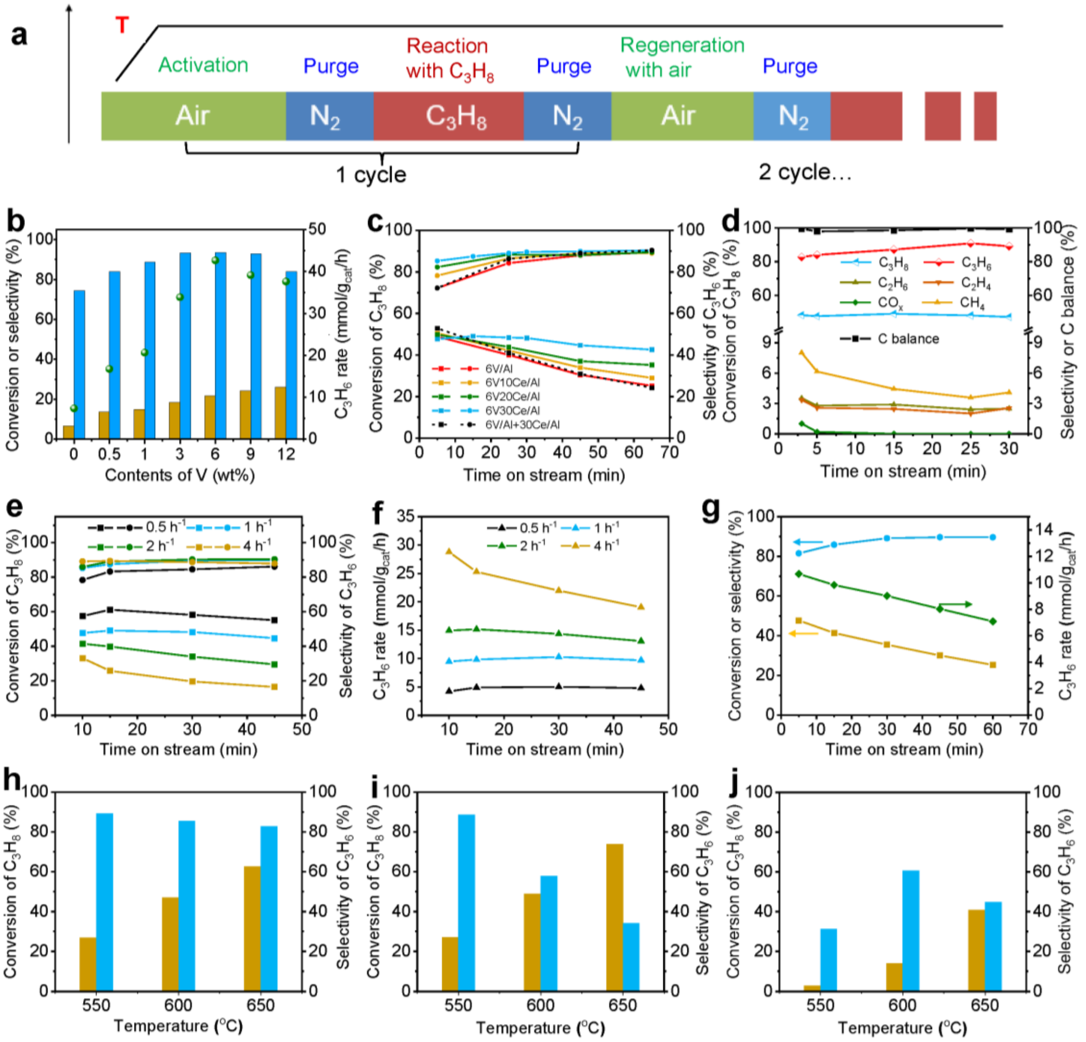

Figure S3 Schematic cyclogram, and effects of V contents, Ce contents, WHSV of propane, and temperature, on propane conversion, propylene selectivity, and formation rates. (**a**) Schematic cyclogram of CL-ODH process. (**b**) Reaction performance as a function of V contents over ceria-vanadia redox catalysts (6V/30CeAl). Reaction conditions: 600 °C, GHSV=25,000 h^{-1}, C_3H_8/N_2=0.25. (**c**) Effect of Ce contents ranging from 0 to 30 wt% and physical mixture of vanadia (6V/Al) and ceria (30CeAl) catalysts (mass ratio 1:1) on propane conversion and propylene selectivity over ceria-vanadia redox catalysts (6V/30CeAl). Reaction test conditions: 600 °C, 1.4 atmospheric pressure, GHSV=2500 h^{-1}, 0.5 g of sample, C_3H_8/N_2 = 0.25. (**d**) Product distribution over ceria-vanadia redox catalysts (6V/30CeAl). Reaction test conditions: 600 °C, 1.4 atmospheric pressure, GHSV=2500 h^{-1}, 0.5 g of sample, C_3H_8/N_2 = 0.25. Effect of WHSV of propane on (**e**) propane conversion, propylene selectivity, and (**f**) propylene formation rates over ceria-vanadia redox catalysts (6V/30CeAl) at the reactor exit. Reaction test conditions: WHSV of propane=0.5, 1, 2,

4 h^{-1} (C_3H_8: 10%, 20%, 40%, 80% vol diluted by N_2), 600 ºC, and 1.4 atmospheric pressure. (**g**) Propane conversion, propylene selectivity, and propylene rate over industrial K-CrO_x/Al_2O_3 catalysts (1 wt.% K, 20 wt.% Cr) measured with reaction time. Reaction test conditions: 600 ºC, 1.4 atmospheric pressure, GHSV=2500 h^{-1}, 0.5 g of sample, C_3H_8/N_2 = 0.25. Effect of temperature on propane conversion and propylene selectivity over (**h**) ceria-vanadia redox catalysts (6V/30CeAl), (**i**) vanadia (6V/Al) and (**j**) ceria (30CeAl) samples. Reaction test conditions: 550, 600 ºC, and 650 ºC, 1.4 atmospheric pressure, GHSV=2500 h^{-1}, 0.5 g of sample, C_3H_8/N_2 = 0.25.

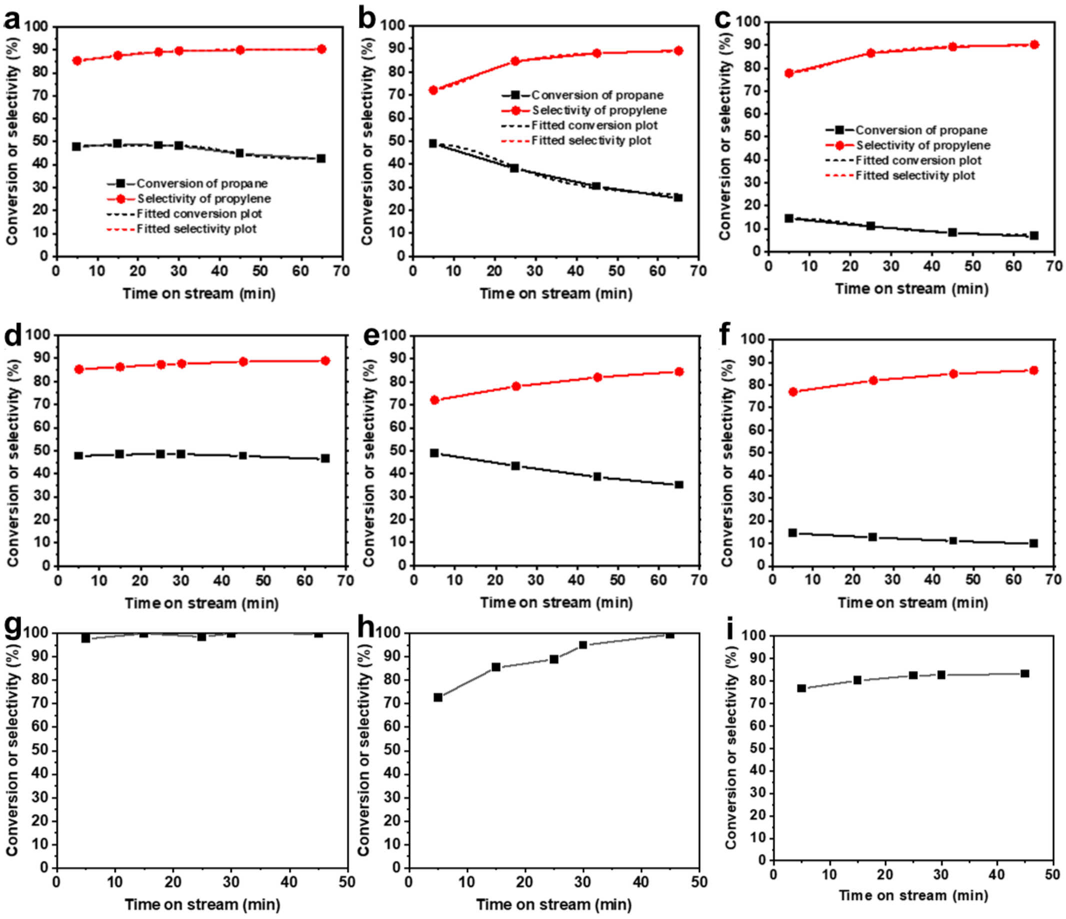

Figure S4 | Reaction performance during the CL-ODH processes. Typical oxidative dehydrogenation product profiles (based on gas phase products) over (a) ceria-vanadia (6V/30CeAl), (b) vanadia (6V/Al), and (c) ceria (30CeAl) samples measured with reaction time. The average conversion and selectivity (based on gas phase products) over (d) vanadia-ceria (6V/30CeAl), (e) vanadia (6V/Al), and (f) ceria (30CeAl) samples were measured with reaction time. The C balance over (g) vanadia-ceria (6V/30CeAl), (h) vanadia (6V/Al), and (i) ceria (30CeAl) samples was measured with reaction time. Reaction test conditions: 600 °C, 1.4 atmospheric pressure, GHSV=2500 h^{-1}, 0.5 g of sample, $C_3H_8/N_2 = 0.25$.

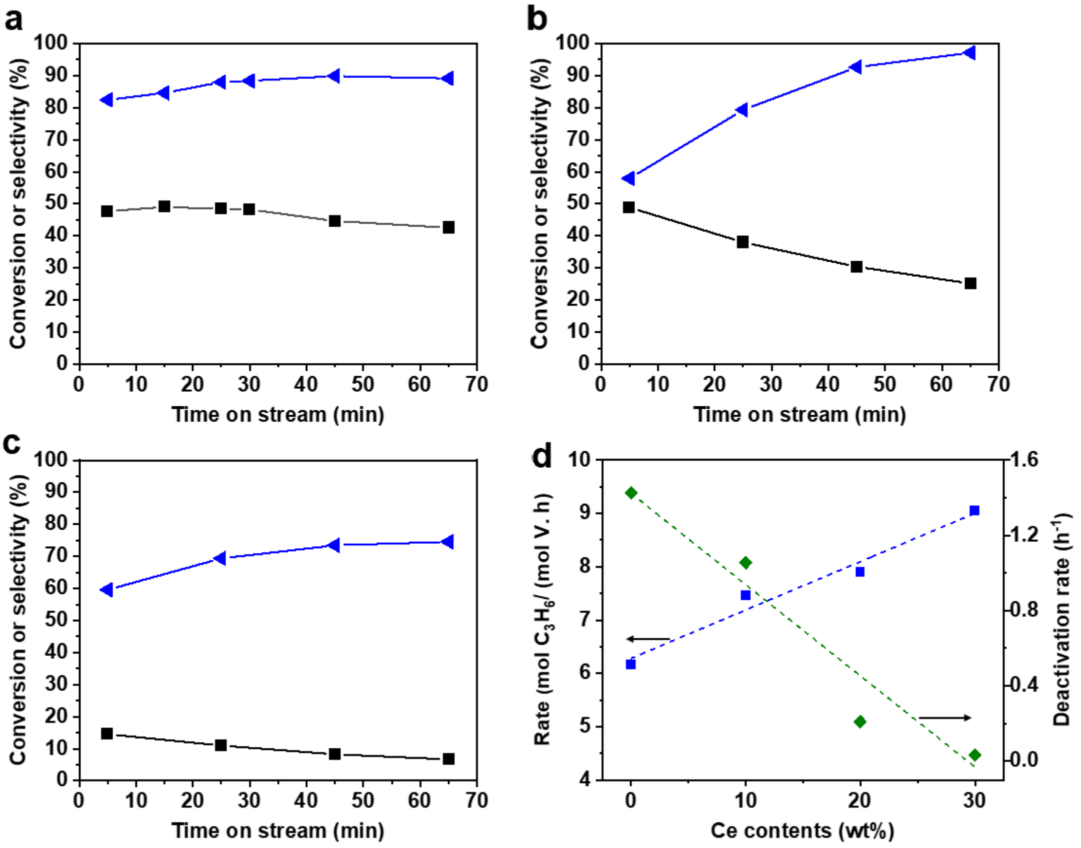

Figure S5 | The propane conversion and propylene selectivity (including coking formation) and the deactivation rates. The propane conversion and propylene selectivity (including coking formation) as a function of the reaction time of (a) ceria-vanadia (6V/30CeAl), (b) vanadia (6V/Al), and (c) ceria (30CeAl) samples measured with reaction time. (d) The deactivation rates as a function of Ce contents over ceria-vanadia catalysts during the 60 minutes. Reaction test conditions: 600 °C, 1.4 atmospheric pressure, GHSV=2500 h^{-1}, 0.5 g of sample, $C_3H_8/N_2 = 0.25$.

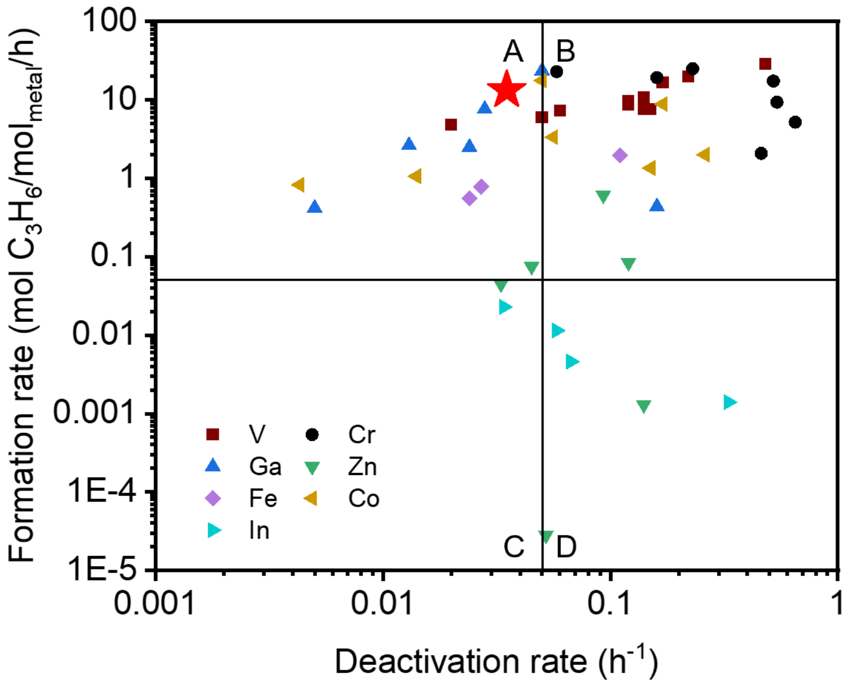

Figure S6 | Comparisons of propylene formation rates as a function of deactivation rate over metal oxide-based catalysts. Normalized activities of the formation rate of C_3H_6 by the specific content of M (Cr, V, Ga, Zr, Zn, Fe, Co, and In) as a function of the deactivation rate for the catalysts described in the literature (see Table S4). A first-order deactivation model is usually used to evaluate the catalyst stability. The formation rate of C_3H_6 is normalized as the unit of mol C_3H_6/mol_{metal}/h. A, B, C, and D represents four different regions with different performance. For example, in region A, higher propylene formation rates and lower deactivation rates are obtained. Comparatively, in region D, lower propylene formation rates and higher deactivation rates are obtained. The details are listed in Table S3.

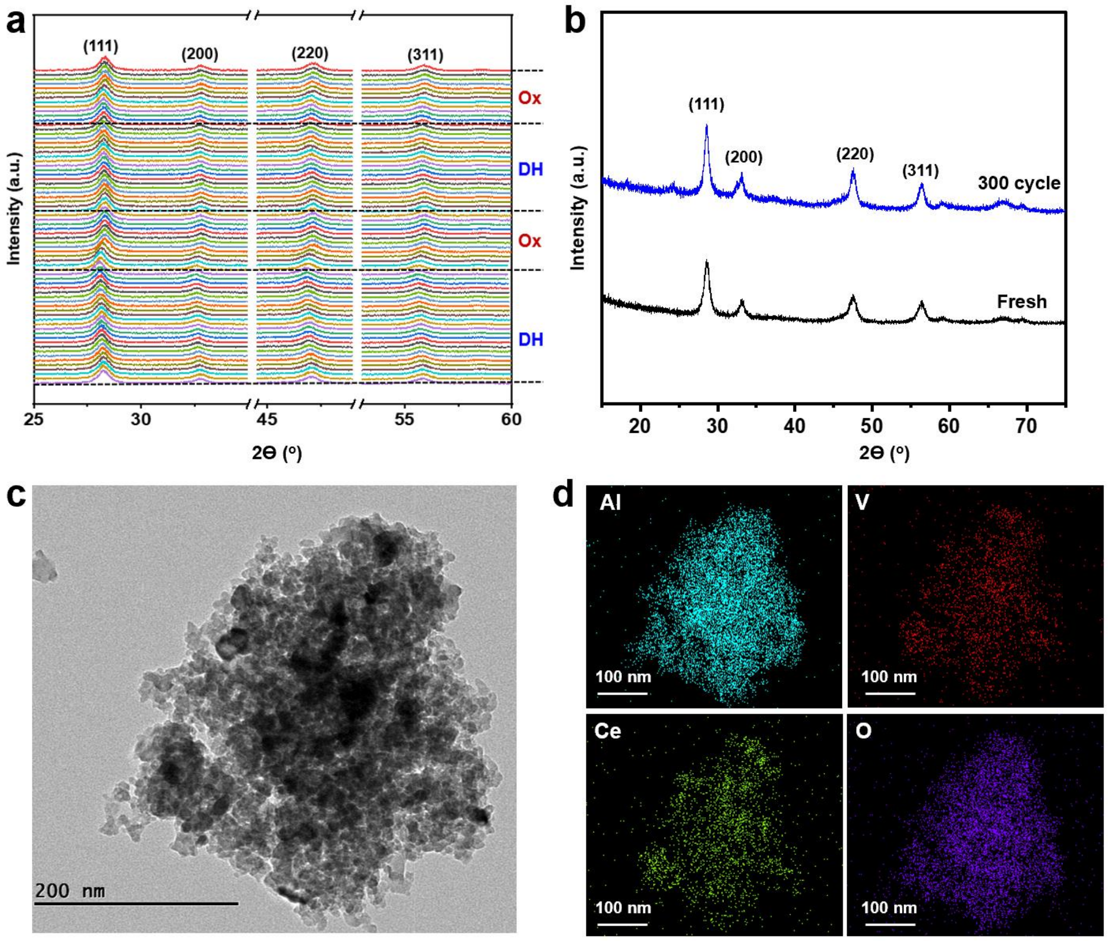

Figure S7 | Structure evolution during the dehydrogenation and redox cycles. (a) *In situ* XRD patterns of ceria-vanadia catalysts (6V/30CeAl) with the reaction time at 600 ºC during the dehydrogenation-oxidation (DH-Ox) cycles. (b) XRD patterns of fresh ceria-vanadia and ceria-vanadia catalysts (6V/30CeAl) after 300 cycles. (c, d) TEM image and EDS-mappings of ceria-vanadia catalysts (6V/30CeAl) after 300 cycles.

There were no significant changes in XRD diffraction patterns after 300 redox cycles, indicating the superior regeneration of crystalline structures. The elements of V and Ce remained uniformly distributed, indicating the superior regeneration of ceria-vanadia catalysts.

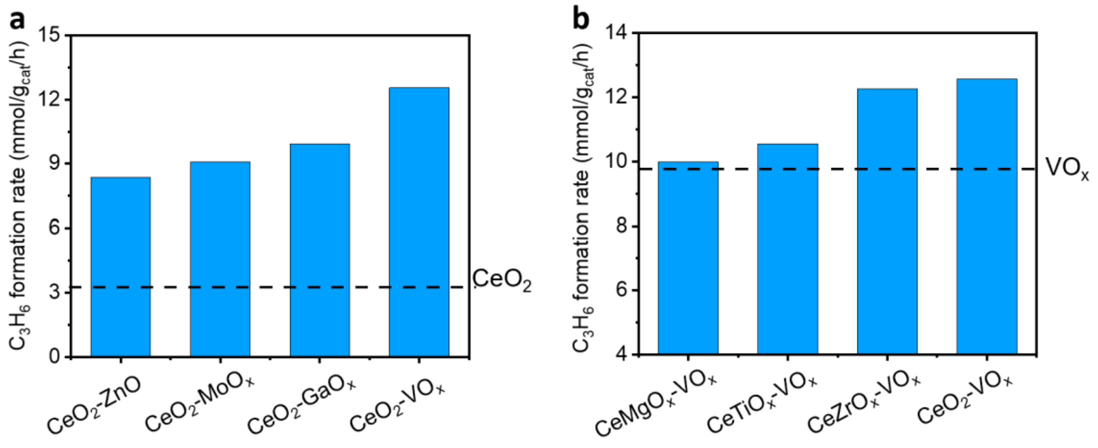

Figure S8 | The propylene formation over heterogeneous oxygen carrier composites. (a) The propylene formation over CeO_2-MO_x: CeO_2-ZnO (6Zn/30CeAl), CeO_2-MoO_x (6Mo/30CeAl), CeO_2-GaO_x (6Ga/30CeAl), and CeO_2-VO_x (6V/30CeAl). (b) The propylene formation over MO_x-VO_x: $CeMgO_x$-VO_x (6V/30Ce3MgAl), $CeTiO_x$-VO_x (6V/30Ce3TiAl), $CeZrO_x$-VO_x (6V/30Ce3ZrAl), and CeO_2-VO_x (6V/30Ce3MgAl). Reaction test conditions: 600 ºC, 1.4 atmospheric pressure, GHSV=2500 h^{-1}, 0.5 g of sample, C_3H_8/N_2 = 0.25.

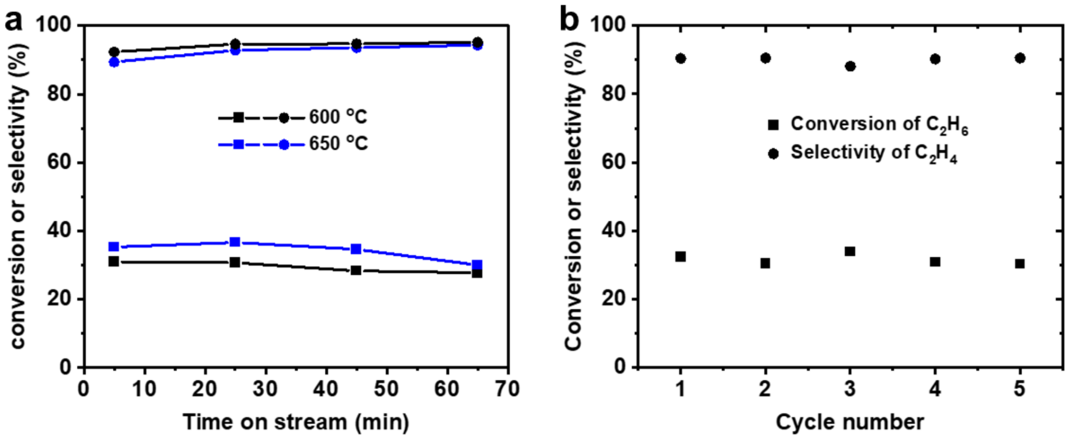

Figure S9 | Reaction performance during the CL-ODH of ethane on ceria-vanadia redox catalysts. (a) The ethane conversion and ethylene selectivity over ceria-vanadia catalysts (6V/30CeAl) were measured with reaction time. Reaction test conditions: 600 °C and 650 °C, 1.4 atmospheric pressure, GHSV=2500 h^{-1}, 0.5 g of sample, C_2H_6/N_2 = 0.25. (b) Cycled scheme and performance over ceria-vanadia. Dehydrogenation step: 600 °C, GHSV =2,500 h^{-1}, C_2H_6/N_2=0.25 for 30 minutes; Inert purge: 650 °C, N_2=40 mL/min for 5 minutes; Oxidation step: 650 °C, 20 vol.% O_2/N_2=20 mL/min for 15 minutes.

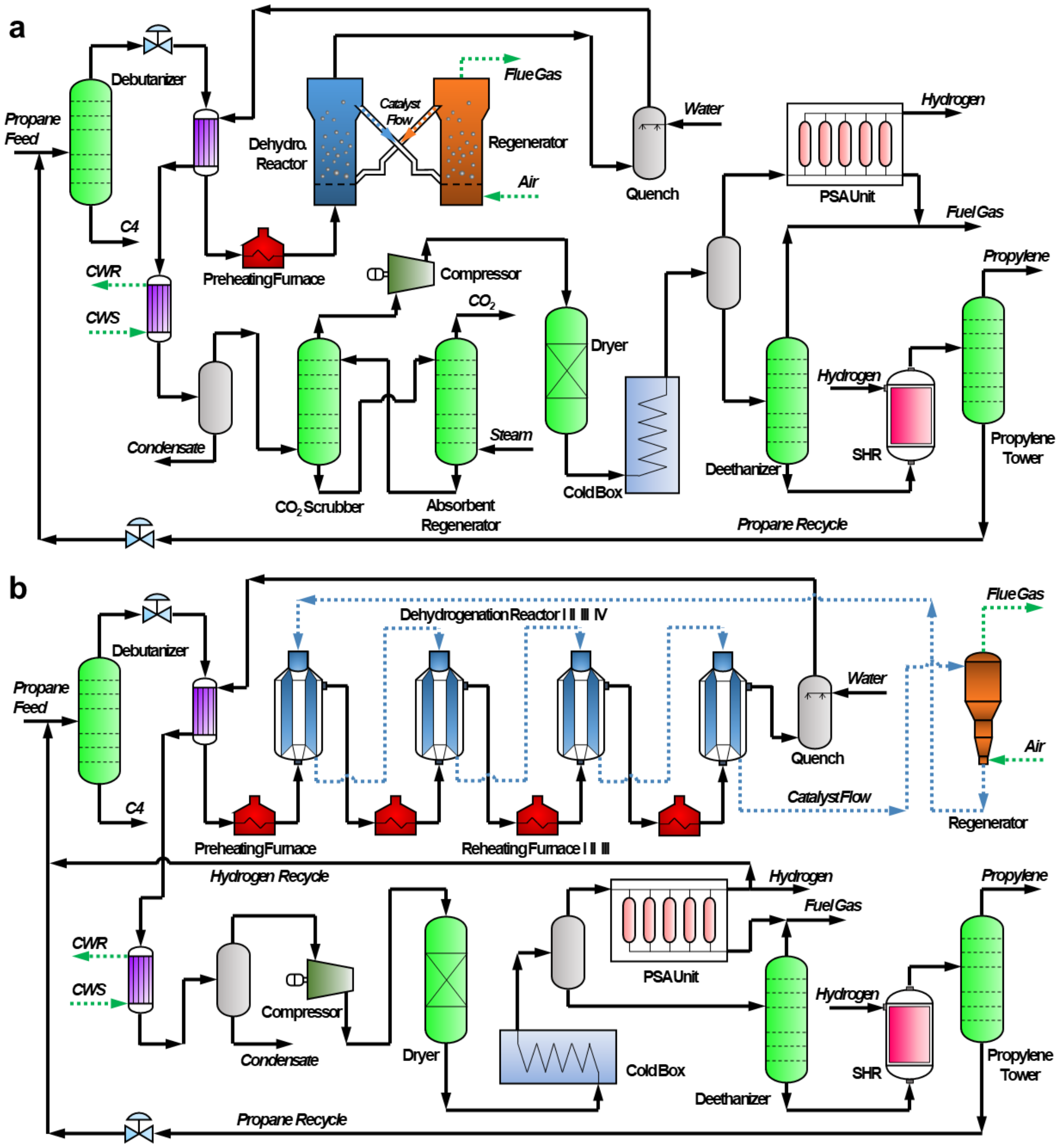

Figure S10 | The scheme of CL-ODH and Oleflex technology by ASPEN Plus simulation. (a) The scheme of CL-ODH technology and the reaction and regeneration of the redox catalyst is isolated spatially or temporally. (b) The scheme of Oleflex technology makes use of 4 adiabatic moving-bed reactors in series to convert paraffin into olefin, which is followed by a continuous catalyst regenerator, and the reaction heat is supplied by the interstage reheating furnace.

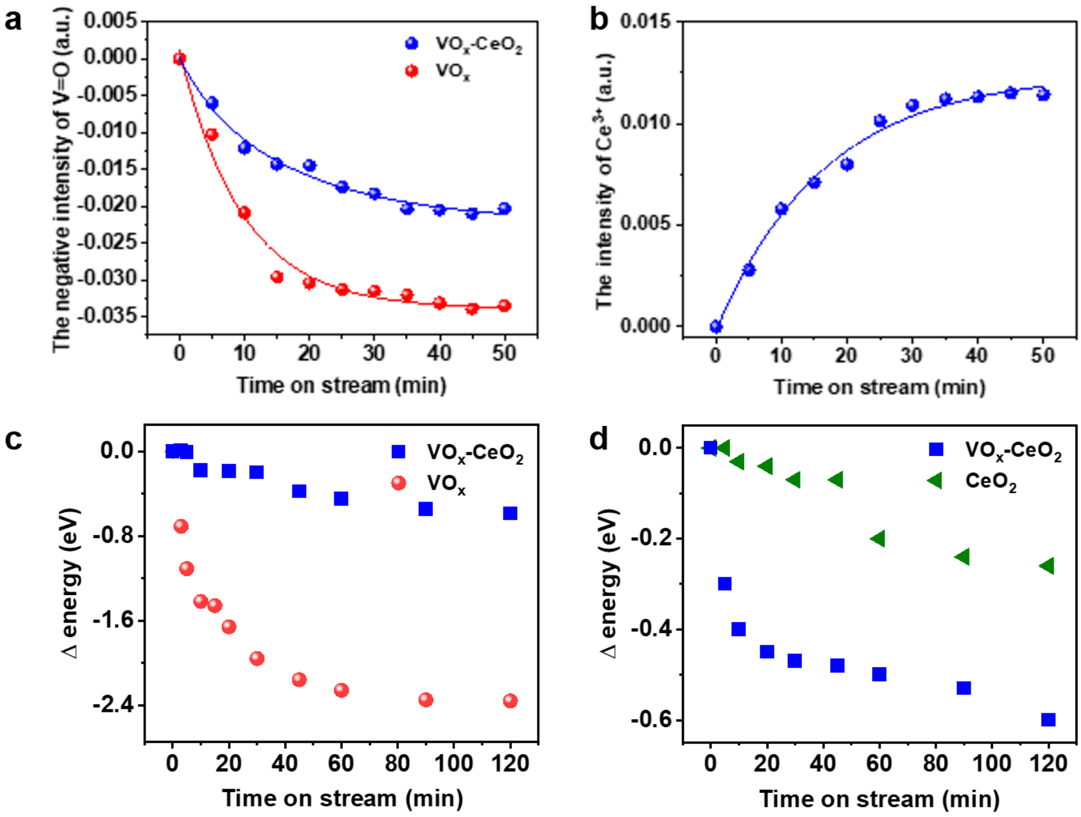

Figure S11 | Experimental evidence of reaction mechanism and the oxygen transfer behavior. (a) The negative intensity of V=O and (b) the positive intensity of Ce^{3+} over vanadia (6V/Al) and ceria-vanadia catalysts (6V/30CeAl) obtained from *in situ* DRIFTS. (c) The calculated energy changes of the white lines of V K-edge in vanadia (6V/Al) and ceria-vanadia catalysts (6V/30CeAl). (d) The calculated energy changes of the white lines of Ce L_3-edge in ceria (30CeAl) and ceria-vanadia catalysts (6V/30CeAl).

For pure VO_x, a more negative V=O band was observed with the reaction time. Comparatively, the V=O band showed no significant change while Ce^{3+} intensity significantly increased, indicating that lattice oxygen from ceria might be spilled to replenish the surface V=O band.

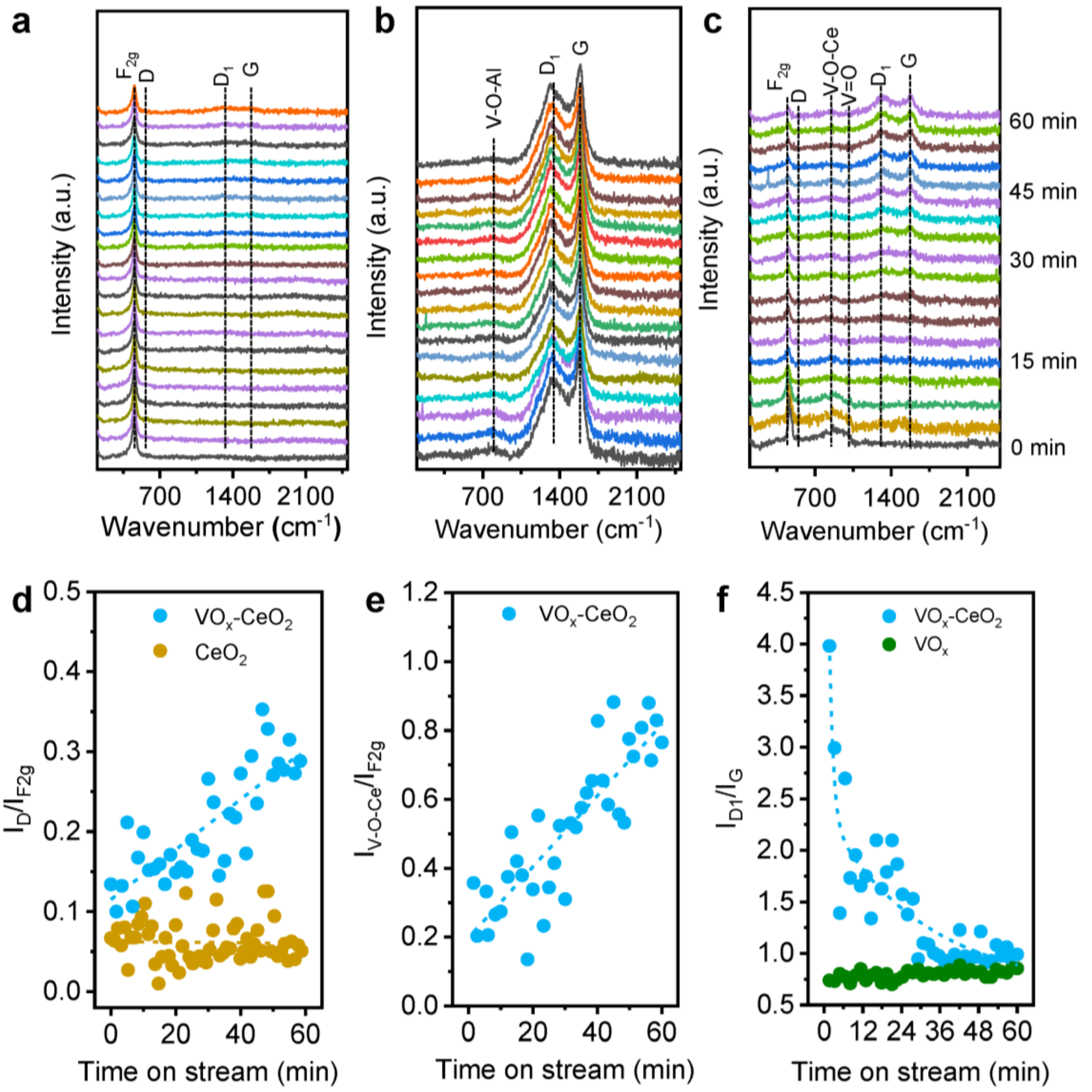

Figure S12 | Experimental evidence of oxygen evolution by *in situ* Raman spectra during propane dehydrogenation. *In situ* Raman spectra during propane dehydrogenation over (a) ceria (30CeAl), (b) vanadia (6V/Al), and (c) ceria-vanadia redox catalysts (6V/30CeAl) at 600 ºC under the atmosphere of 20% C_3H_8/N_2 (20 mL/min). The calculated intensity ratios of (d) *D* mode and F_{2g} bands of CeO_2, (e) F_{2g} and V-O-Ce bands, and (f) D_1 and G obtained from *in situ* Raman spectra over (a) ceria (30CeAl), (b) vanadia (6V/Al), and (c) ceria-vanadia redox catalysts (6V/30CeAl).

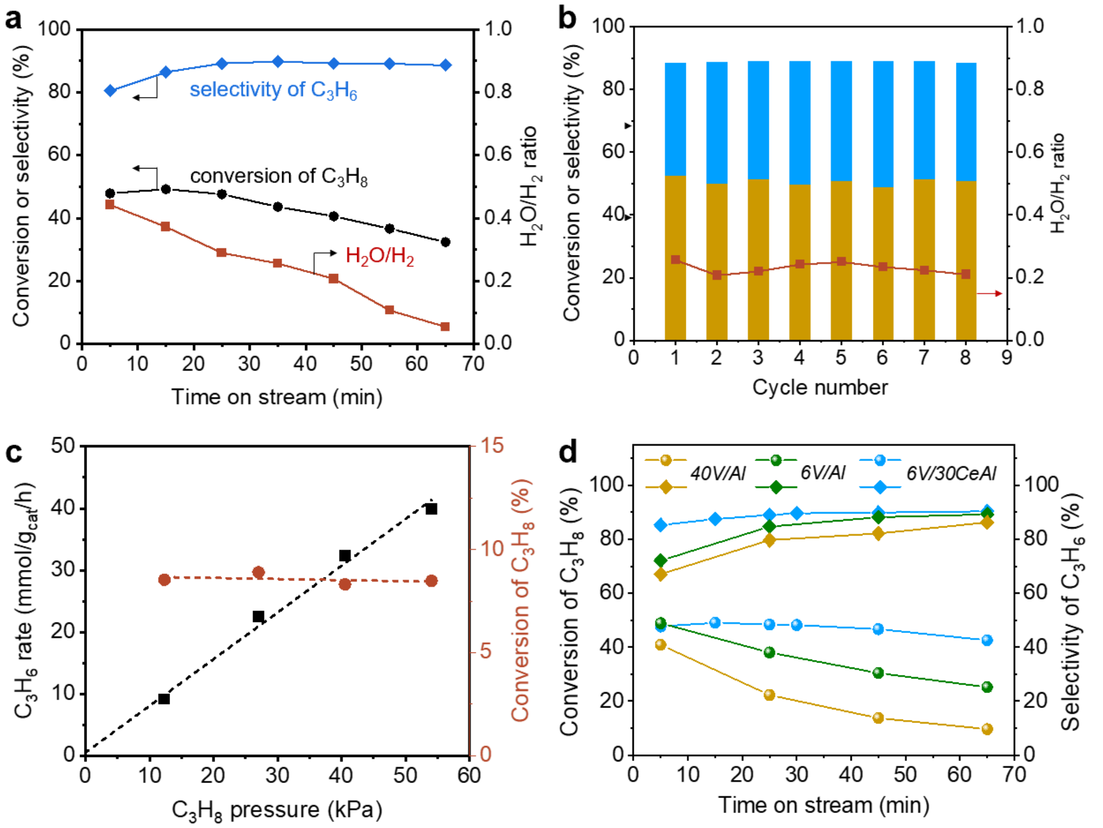

Figure S13 | The formation of H_2O and H_2, and C_3H_6 formation rates. (a) Propane conversion, propylene selectivity and ratios of H_2O/H_2 as a function of time on stream over ceria-vanadia redox catalysts (6V/30CeAl). Conditions: 600 °C, GHSV =2,500 h^{-1}, C_3H_8/N_2=0.25. (b) Cyclic performance over ceria-vanadia redox catalysts (6V/30CeAl). (Dehydrogenation step: 600 °C, GHSV =2,500 h^{-1}, C_3H_8/N_2=0.25 for 30 minutes; Inert purge: 600 °C, N_2=40 mL/min for 5 minutes; Oxidation step: 600 °C, 20 vol.% O_2/N_2=20 mL/min for 15 minutes). (c) C_3H_6 formation rates and C_3H_8 conversion as a function of C_3H_8 pressure (diluted by N_2) at 600 °C. (d) C_3H_8 conversion and C_3H_6 selectivity as a function of time on 40V/Al, 6V/Al and 6V/30CeAl at 600 °C. Reaction test conditions: 600 °C, 1.4 atmospheric pressure, GHSV=2500 h^{-1}, 0.5 g of sample, C_3H_8/N_2 = 0.25.

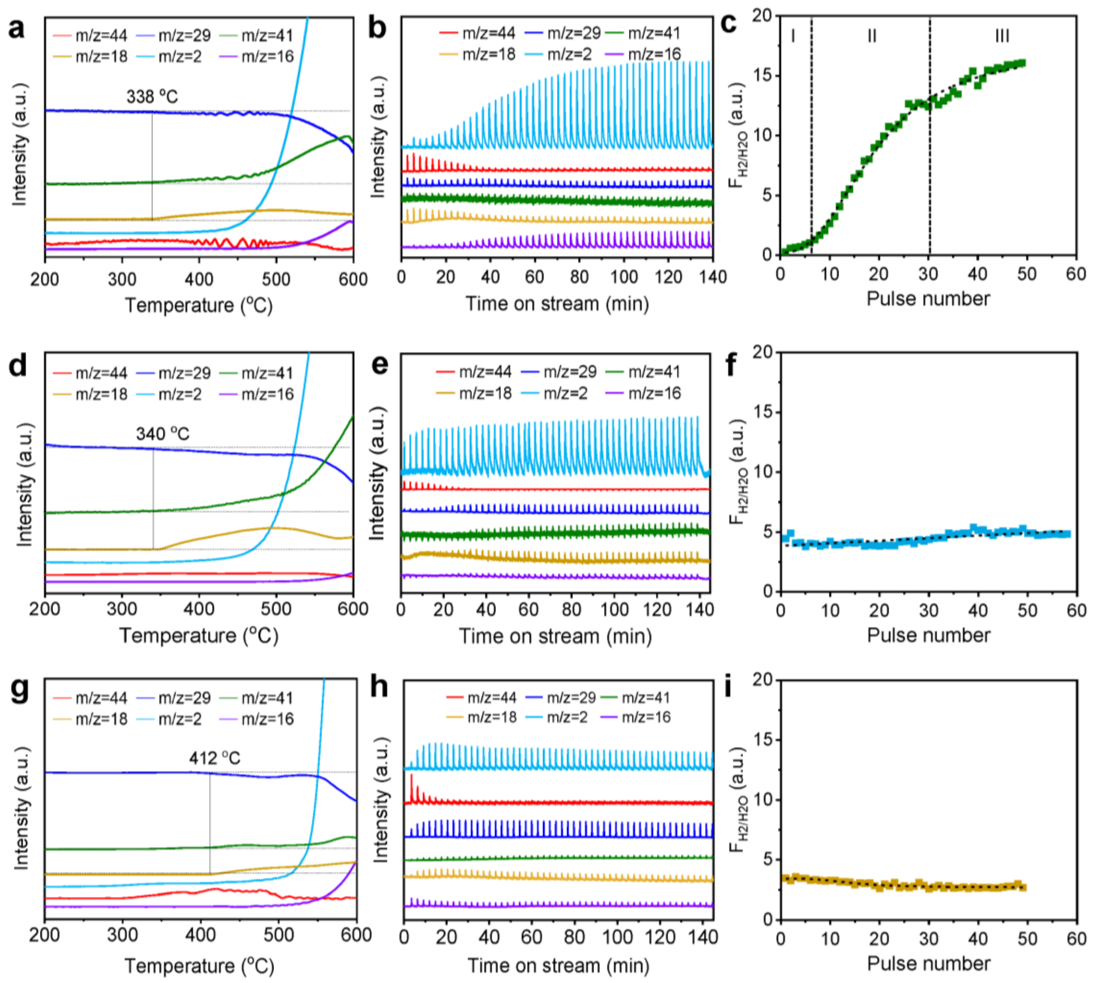

Figure S14 | Surface reaction routes determined by the temperature-programmed surface reaction and transient pulse experiments. C_3H_8-temperature programmed surface reaction (TPSR), Product distributions at 600 °C and the calculated $F_{H2/H2O}$, which is the intensity ratio of H_2/H_2O obtained from the pulse profiles, (a-c) vanadia (6V/Al), (d-f) ceria-vanadia (6V/30CeAl) and (g-i) ceria (30CeAl) catalysts. C_3H_8, C_3H_6, CO_2, CH_4, H_2, and H_2O, m/e equals 29, 41, 44, 16, 2 and 18, respectively.

a

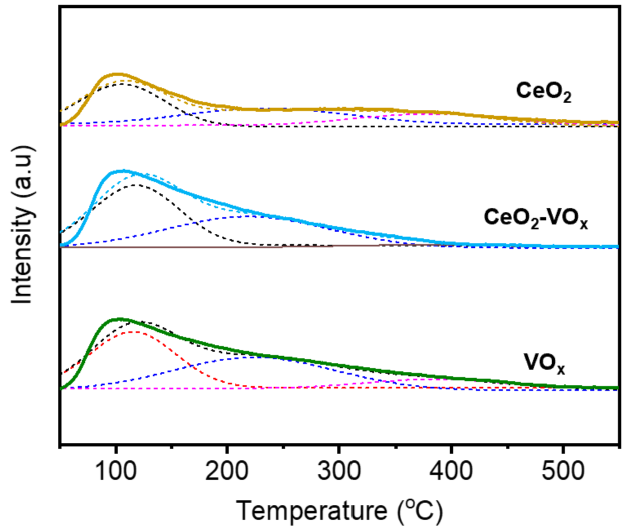

b

Fitted results of NH_3-TPD experiments of ceria, vanadia and ceria-vanadia redox catalysts.

Samples	T_M (°C)			Total area (a.u.)	Percent of peak area (%)		
	I	II	III		I	II	III
CeO_2	104	231	392	44.0	44.3	33.9	21.7
CeO_2-VO_x	102	231	392	59.1	47.8	48.7	3.5
VO_x	99	231	392	64.5	41.1	47.3	11.5

Figure S15 | (a) **NH_3-TPD profiles and (b) fitted results of ceria (30CeAl), vanadia (6V/Al), and ceria-vanadia redox catalysts (6V/30CeAl).**

The NH_3 desorption in the temperature regions of 120−200 °C, 200−350 °C, and 350−450 °C were regarded as the weak, medium, and strong acid sites, respectively. To obtain the semiquantitative results of total acidity and the distribution of acidic strength, we sued a Gaussian peak fitting method to deconvolute the NH_3-TPD curves.

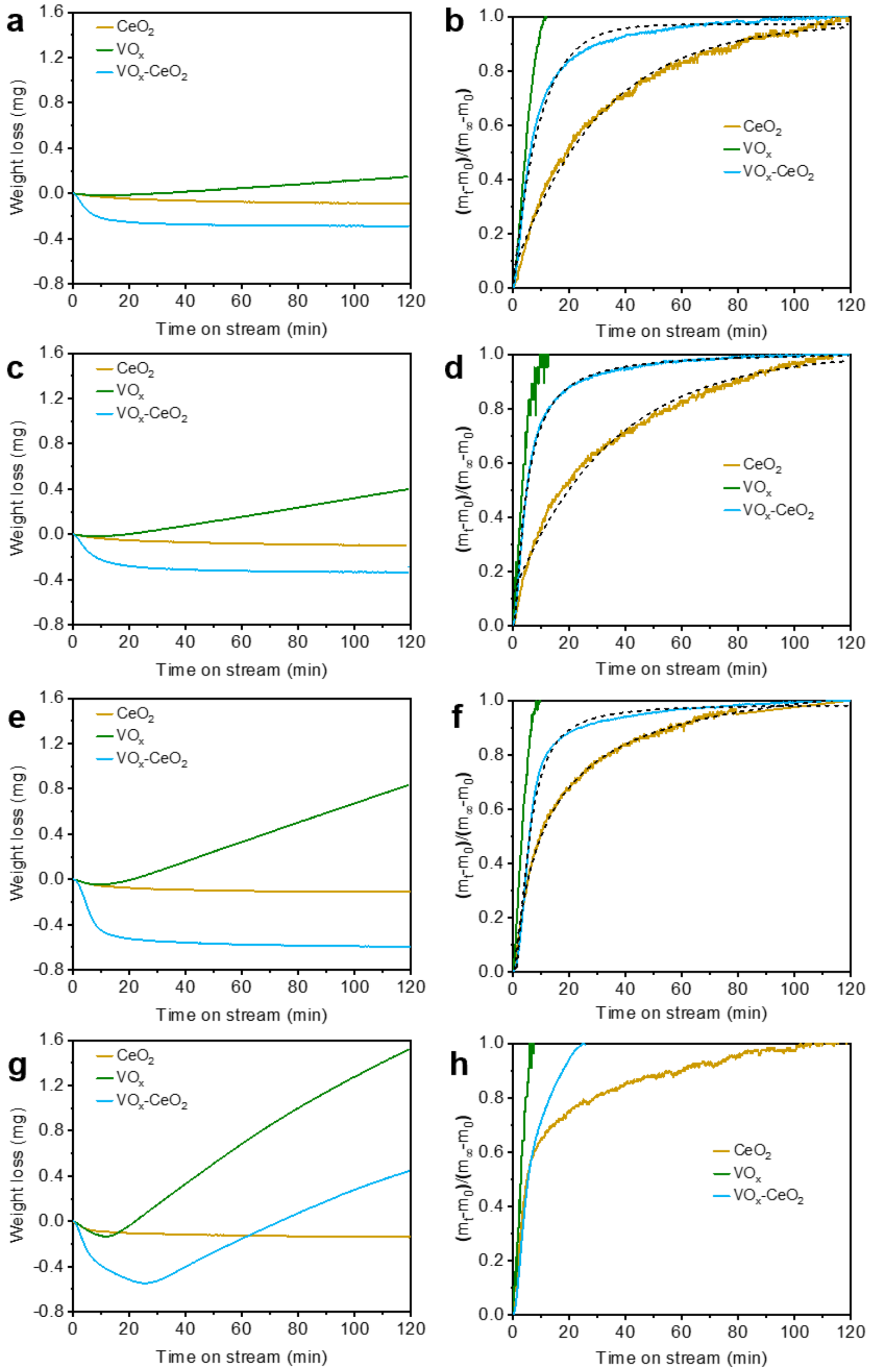

Figure S16 | Oxygen release kinetics determined by thermogravimetry relaxation experiments. Isothermal time dependence of the mass loss and relaxation curve in the form of fractional weight: (a, b) at 550 ºC, (c, d) 575 ºC, (e, f) 600 ºC, and (g, h) 625 ºC in a mixture of 20% C_3H_8/He (10 mL/min) for CeO_2 (30CeAl), VO_x (6V/Al) and VO_x-CeO_2 (6V/30CeAl). The dash lines represent the fitted thermogravimetry relaxation curves.

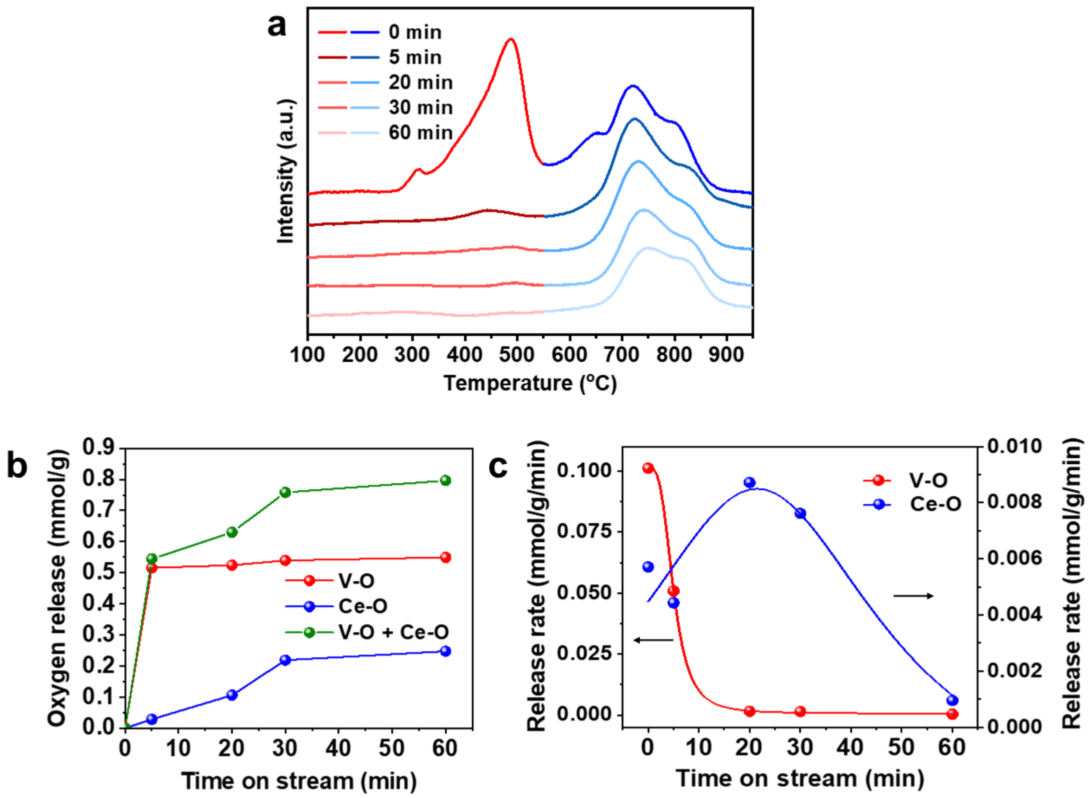

Figure S17 | The determined consumption amount and rates of various oxygen species. (a) H_2-TPR profiles of ceria-vanadia catalysts (6V/30CeAl) after H_2 pre-reduction at 600 °C for 0-60 minutes. (b) The calculated oxygen release amount is ascribed from vanadia (6V/Al), ceria (30CeAl), and ceria-vanadia (6V/30CeAl), respectively. (c) The calculated oxygen release rates are ascribed from vanadia (low temperature reduction peak from 200-550 °C) and ceria (high-temperature reduction peaks from (550-950 °C), respectively.

At the initial period before 5 minutes, V-O species were ready to be consumed. However, when V-O species were completely consumed, Ce-O species sped up to be consumed, and it reached be quickest consumption rate at about 20th min with a rate of 0.094 mmol/g/min. The total oxygen release amount was determined to be ~0.8 mmol/g during the 60 min-H_2 pre-reduction.

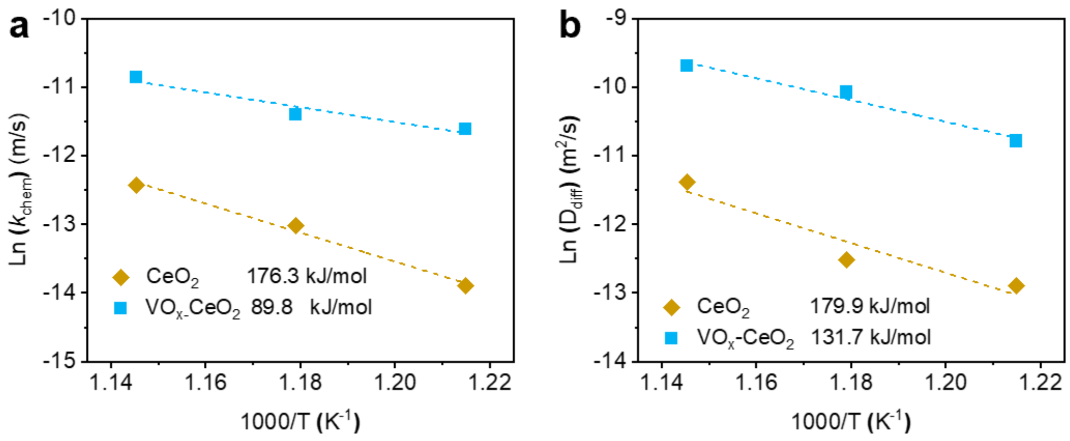

Figure S18 | The Arrhenius plots of k_{chem} and D_{diff} from 550 to 600 °C. Arrhenius plots of the (a) k_{chem} for CeO_2 (30CeAl) and VO_x-CeO_2 (6V/30CeAl) and (b) D_{diff} for CeO_2 (30CeAl) and VO_x-CeO_2 (6V/30CeAl) over a temperature range from 550 to 600 °C.

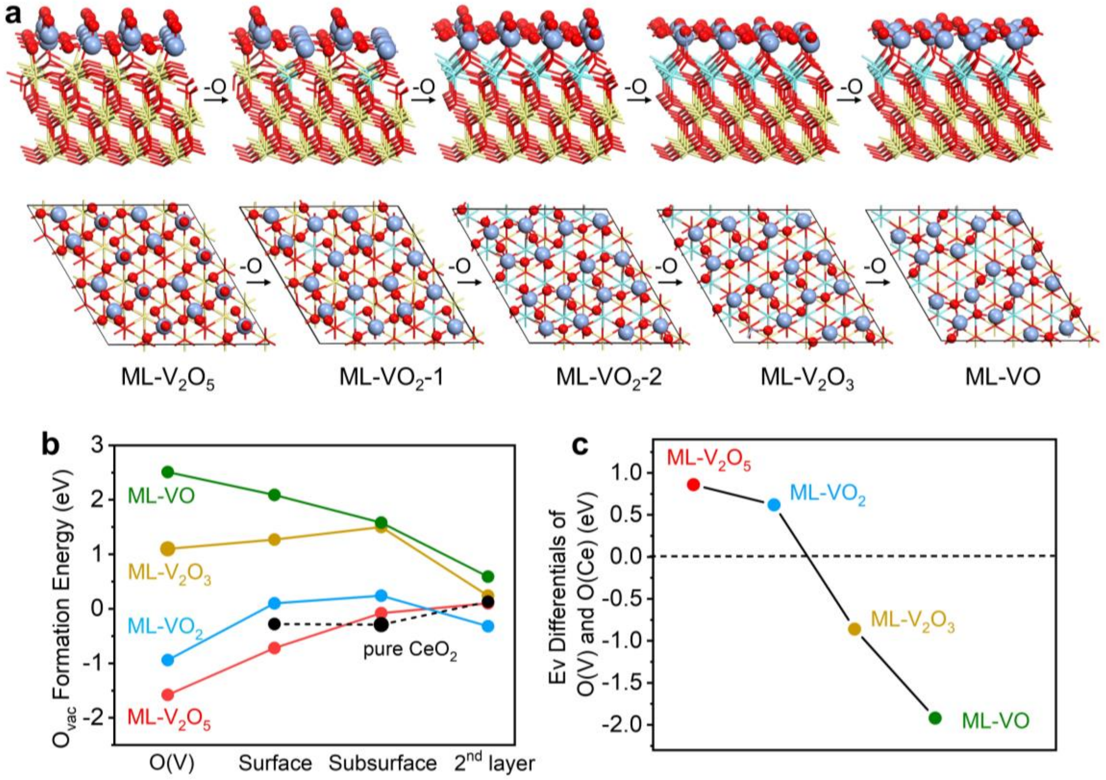

Figure S19 | Reduction process and relative oxygen formation energies derived by DFT calculations. (a) Models of ML-VO_x on CeO_2 with different V/O ratios. V: dark blue; Ce^{4+}: yellow; Ce^{3+}; bright blue; O: red. (b) O_{vac} formation energies in the continuous models with different V/O ratios upon O elimination: ML-V_2O_5, ML-VO_2, ML-V_2O_3, and ML-VO. (c) O_{vac} formation energy difference of O(V) and O(Ce) in each model.

The ML-V_2O_5 and ML-VO_2-2 models are obtained according to the calculation of J. Sauer et al. *(42)*, which are global minimum structures. Other models are established by constructing oxygen vacancies that are easiest to form in VO_x (most negative oxygen vacancy formation energy) in these two models. The trend that VO_x loses O while Ce is reduced is clear according to our calculation. The sites of Ce^{3+} centers are marked in the figure. The calculated oxygen vacancies formation energy for sites in these models are shown in (b) and (c).

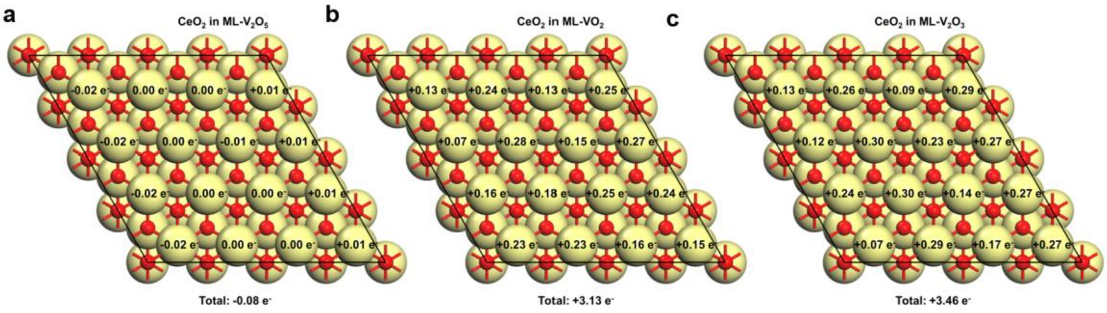

Figure S20 | Bader charge analysis. Bader charge difference for CeO_2 in (a) ML-V_2O_5, (b)ML-VO_2 and (c) ML-V_2O_3. The Bader charge result for pure CeO_2 surface is taken as reference to show how electrons accumulate in ML-VO_x.

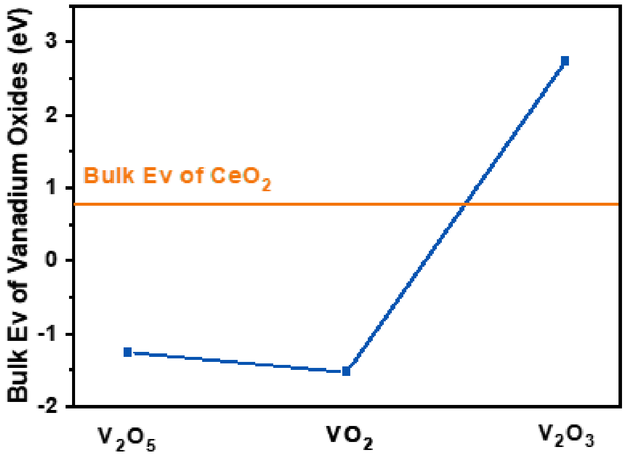

Figure S21 | O$_{vac}$ formation energies of bulk V_2O_5, VO_2, V_2O_3, and CeO_2.

Oxygen formation energies in the bulk of several materials are shown to denote the potential oxygen supply ability. For vanadia, V_2O_5 and VO_2 show typical overoxidation features, so their oxygen vacancy formation energies are too negative. Meantime, V_2O_3 is an active constituent for non-oxidative dehydrogenation, so its oxygen vacancy formation energy is too positive, which means V_2O_3 is nonreducible and cannot act as an oxygen carrier. Hence a proper oxygen carrier for CL-ODH needs to balance between these two extremes, which is the CeO_2 in the figure and has the potential to act as a proper oxygen carrier in our application.

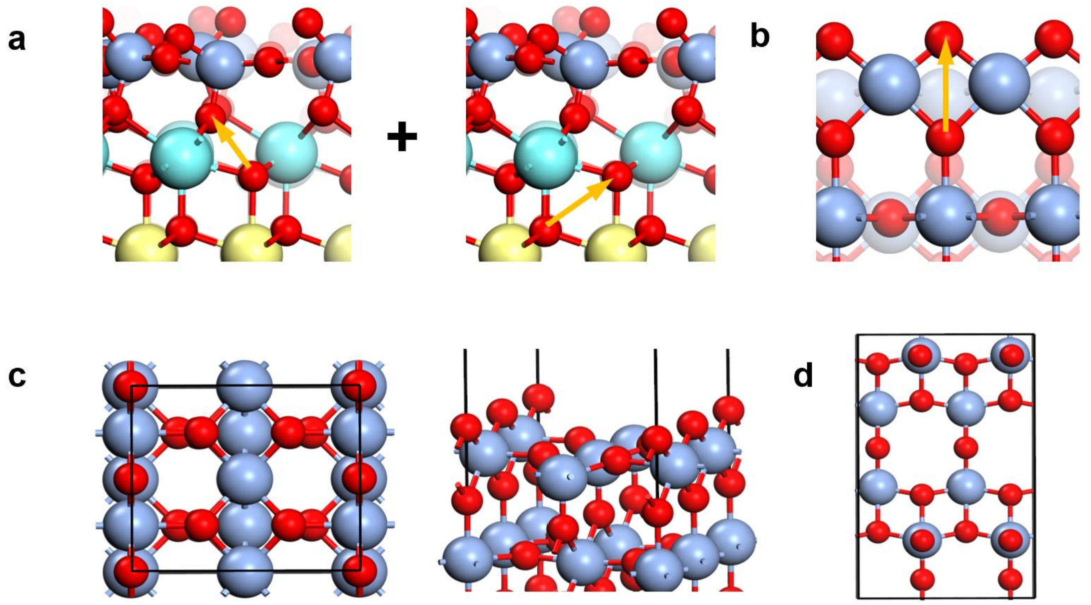

Figure S22 | Oxygen diffusion pathway derived by DFT calculations. (a) Isolated hopping in CeO_2 and corresponding barrier over ceria-vanadia. (b) Oxygen diffusion over crystalline VO_2. (c) Models of crystalline VO_2. (d) Model of crystalline V_2O_5. V: dark blue; Ce^{4+}: yellow; Ce^{3+}; bright blue; O: red.

Isolated hopping is comprised of two hopping processes that happen step-by-step, while corresponding concerted hopping means such two hopping processes happen simultaneously and reach the same final structure. According to our calculation, such isolated hopping is difficult to proceed kinetically due to the high barrier (1.47 eV).

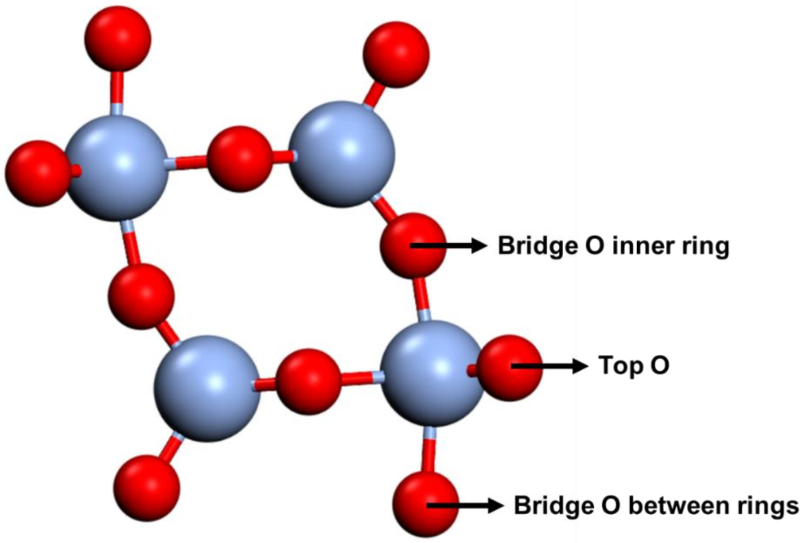

Figure S23 | Unit [VO_4] rings in ML-VO_2.

Unit [VO_4] rings in ML-VO_2 with three kinds of O sites: Top O (O(I)), bridge O inner ring (O(II)), and bridge O between rings (O(III)). For the four V atoms, the coordination number of the two V atoms with top O is five, while the coordination number of the other two V atoms is four.

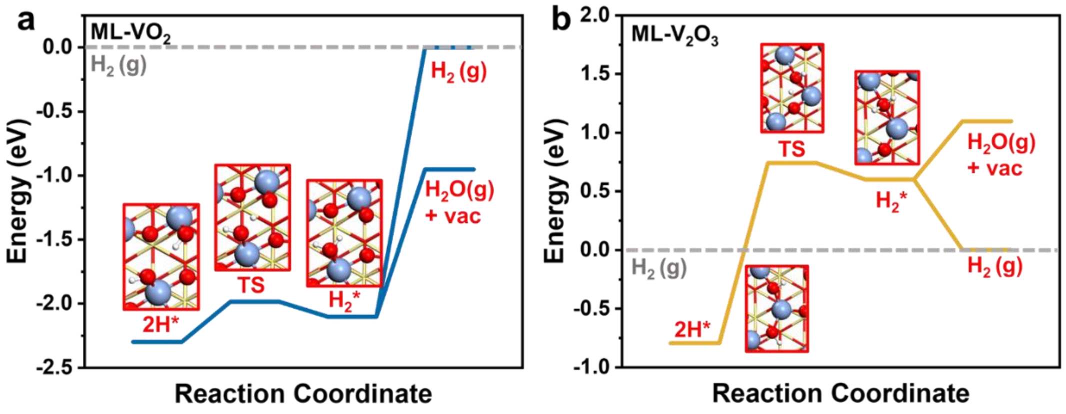

Figure S24 | Reaction energy profiles of H_2O formation and H_2 formation over (a) ML-VO_2 and (b) ML-V_2O_3.

The result indicates in ML-VO_2 period, the main adsorption sites for H is V=O site. The activation barrier of formation H_2* intermediate is low and the formed H_2* has a strong preference to form H_2O(g) rather than H_2(g). Such results denote ML-VO_2 is still in the CL-ODH dominant period. When the vanadia is further reduced, the model enters ML-V_2O_3 period. In Figure S13 (b), we could observe all V=O is removed, and main adsorption site becomes V-O-V. The formation activation barrier of H_2* is higher compared with the barrier in ML-VO_2, indicating the elimination of H* and regeneration of active site are more difficult. Further, H_2* tends to form H_2(g) in this period, denoting the model already enters PDH dominant period.

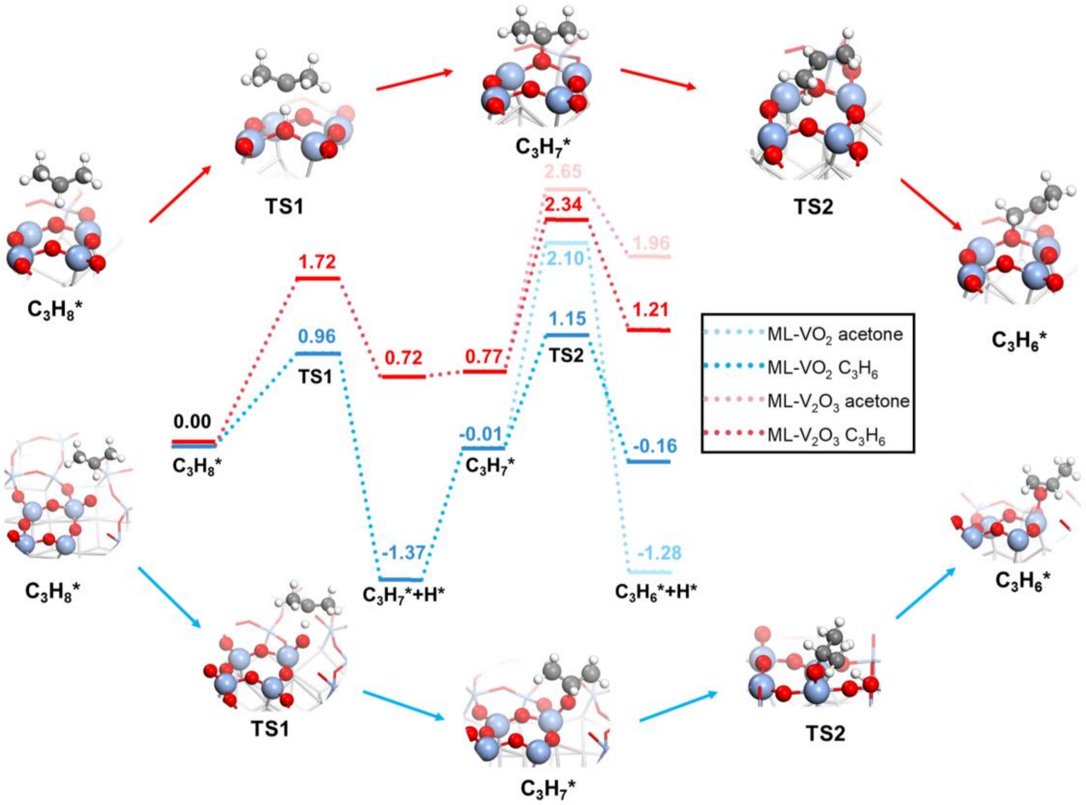

Figure S25 | Calculated potential energy diagram. The Calculated potential energy diagram for [V_4O_8] in ML-VO_2 (blue) and [V_4O_6] in ML-V_2O_3 (red). Reaction steps include (i) dehydrogenation from propane to absorbed propyl. (ii) dehydrogenation absorbed propyl to absorbed propene. (iii) dehydrogenation from absorbed propyl to acetone (byproduct).

According to the calculation, the top site (V=O) in [V_4O_8] unit structure of ML-VO_2 showed optimal reaction performance from perspectives of both activity and selectivity. Compared to the bridge site (V-O-V) in [V_4O_6] of ML-V_2O_3, V=O has lower activation barriers in the first and second dehydrogenation step of propane but higher barriers in the acetone production reaction.

Table S1. Physicochemical properties of vanadia-ceria redox catalysts with different V loadings.

Sample	BET (m^2/g)	V weight (%)[a]	Vanadia surface density (V/nm^2)[b]
6V/Al	152	5.4	4.2
30Ce/Al	115.9		-
0.5V/30CeAl	117.3	0.4	0.4
1V/30CeAl	116.7	0.8	0.7
3V/30CeAl	130.3	2.1	1.9
6V/30CeAl	112.6	4.1	4.3
9V/30CeAl	108.7	5.9	6.4
12V/30CeAl	100.6	7.6	8.9

[a] The vanadia weight is determined by inductively coupled plasma optical emission spectroscopy (ICP-OES).

[b] The vanadia surface density is calculated according to the number of V atoms and the BET of the supports.

Table S2. XPS quantification of V^{5+}, V^{4+}, V^{3+}, Ce^{4+}, and Ce^{3+} of vanadia (6V/Al), ceria (30CeAl), and vanadia-ceria catalysts (6V/30CeAl).

**Samples**	**V^{5+} (%)**	**V^{4+} (%)**	**V^{3+} (%)**	**Ce^{3+} (%)**	**Ce^{4+} (%)**
VO_x	16	45	39	NA	NA
VO_x-CeO_2	18	44	38	41	59
CeO_2	NA	NA	NA	30	70

Table S3. Comparison of VO_x-CeO_2 catalysts with state-of-the-art ODH, PDH and CL-ODH catalysts.

Number	Sample	Temp (°C)	Model	Ratio (C_3H_8:O_2)	P_{C3H8} (kPa)	C_3H_8 Conv. (%)[b]	C_3H_6 sel. (%)[b]	Ref.
1	$PtPd/Al_2O_3/SiO_2$	450	ODH	2:1	10	32.4	17.0	1
2	Pd/MgVO	450	ODH	3.5:1	14	25.0	32.0	2
3	BS-1	570	ODH	1:1	16.6	43.7	53.3	3
4	BNNT	490	ODH	2:1	30	16.0	~75.0	4
5	Cr_2O_3/SBA-15	450	ODH	1:1	2.5	29.7	16.0	5
6	MoO_x/Al_2O_3	500	ODH	1:1	4	12.9	27.0	6
7	MoO_x/Al_2O_3	550	ODH	1:1	4	32.4	27.3	6
8	VO_x/SBA-15	600	ODH	2:1	10	19.6	41.0	7
9	VO_x/Al_2O_3	500	ODH	2:1	10	~18	~40	8
10	$VO_x/TiO_x/SiO_2$	500	ODH	2:1	~10	9.5	50.0	9
11	VO_x/ZrO_2	500	ODH	1:1	~10	33.1	17.1	10
12	VO_x/graphene	500	ODH	2:1	16.7	5.0	92.1	11
13	$(Pt/Al_2O_3)@35cIn_2O_3$	450	ODH	2:1	10	40	75.6	12
14	CrO_x/SBA-1	550	PDH	-	6.7	37	85	13
15	Cr-Al-800	600	PDH	-	101	33.2	90.4	14
16	$Cr_{10}Zr_{90}O_x$	550	PDH	-	40.4	30	82	15
17	VO_x/γ- Al_2O_3	600	PDH	-	~39	25	72	16
18	VO_x/Si_{Beta}	600	PDH	-	5	38	88	17
19	VO_x/MCM-41	550	PDH	-	30.4	22	92	18
20	$VO_x/(SiO_2+Al_2O_3)$	550	PDH	-	36	31	80	19
21	$VO_x/(SiO_2+Al_2O_3)$	550	PDH	-	48	35	80	20
22	VO_x-K/meso-Al_2O_3	610	PDH	-	28	33	83	21
23	VO_x/γ- Al_2O_3	600	PDH	-	28	32	94	22
24	$PtSn/MgAl_2O_4$	580	PDH	-	10	46.4	99.5	23
25	PtSn/MgSBA-15	580	PDH		70	43	97.8	24
26	$PtSn/Al_2O_3$(A750)	590	PDH		16	49	97	25
27	Pt/Sn-Beta	570	PDH		10	50.5	92.5	26
28	Pt–Sn-ZSM	550	CL-ODH	-	~101	~23	98	27
29	V-Mo-O oxide	500	CL-ODH	-	28.4	~38	~80	28
30	$LaNiO_x$	550	CL-ODH	-	10.1	11	40	29
31	VO_x/CaO-γAl_2O_3	640	CL-ODH	-	-	25	94	30
32	CeO_2/Al_2O_3	600	CL-ODH	-	28.4	10.5[a]	81[a]	This work
33	VO_x/Al_2O_3	600	CL-ODH	-	28.4	36[a]	78[a]	This work

34	VO_x/CeO_2-Al_2O_3 redox catalyst	600	CL-ODH	-	28.4	49[a]	89[a]	This work

[a] Propane conversion and propylene selectivity during the dehydrogenation step.

[b] Initial propane conversion and propylene selectivity.

Table S4. Summary of the catalytic data of representative oxide-based catalysts used in propane dehydrogenation.

No.	Catalysts[a]	Temp /°C	WHSV /h^{-1}	Components /%	Conversion /%	Selectivity /%	Lifetime /h	Formation rate[b] /mol $C_3H_6 \cdot mol_M^{-1} \cdot h^{-1}$	k_d [c] /h^{-1}	Ref.
1	4.3wt%VO_x/MCM-41	550	1.78	C_3H_8=40, N_2=60	22-16	92-93	100	4.8	0.02	18
2	4.3wt%VO_x/(10wt%$SiO_2$90wt%Al_2O_3)	550	1.78	C_3H_8=40, N_2=60	31-11	80-94	60	6.0	0.05	19
3	4.2wt%VO_x/(10wt%$SiO_2$90wt%Al_2O_3)	550	0.94	C_3H_8=40, N_2=60	35-12	80-95	25	7.3	0.06	20
4	10wt%VO_x-0.1wt%K/meso-Al_2O_3	610	2.85	C_3H_8=80, N_2=20	70-25	87-83	40	20.0	0.22	31
5	12wt%VO_x/γ- Al_2O_3	600	3.30	C_3H_8=28, H_2=28, N_2=44	32-16	~94	NA	9.6	0.12	32
6	1.4wt%VO_x/MCM-41	550	0.57	C_3H_8=40, N_2=60	<10	87-95	22.5		0.18	33
7	12wt%VO_x-1wt%MgO/γ- Al_2O_3	600	3.30	C_3H_8=28, H_2=28, N_2=44	33-24	83-91	NA	8.7	0.12	21
8	5.26wt%VO_x/SiO_2	580	0.59	C_3H_8=9.1, Ar=90.9	65-45	~90	32	7.6	0.14	34
9	3.7wt%VO_x/SiO_2	580	0.59	C_3H_8=9.1, Ar=90.9	65-43	~90	36	10.8	0.14	35
10	2.9wt%VO_x/γ- Al_2O_3	600	0.88	C_3H_8=7.5, Ar=92.5	51-22	~93	8	16.8	0.17	36
11	6wt%VO_x/γ- Al_2O_3	600	8.25	C_3H_8=28, H_2=28, N_2=72	25-15	72-80	NA	28.7	0.48	16

12	3wt%VO_x/SiBeta	600	0.59	C_3H_8=5, N_2=95	38-23	88-94	16	7.7	0.15	17
13	mesoporous Cr_2O_3/Al_2O_3(9wt% Cr)	580	8.63	C_3H_8=5, He=95	15.7-10.0	98	1	17.4	0.52	37
14	20wt%Cr/CMK-3	550	1.2	C_3H_8=6.7, He=93.3	47.4-11	84.7-95	3.67	9.4	0.54	38
15	7.5wt%Cr/Al_2O_3	600	2.16	C_3H_8=20, Ar=80	63-25.7	88-89	10	19.2	0.16	39
16	2.5wt%Cr-5wt%Ni/Al	550	1.08	C_3H_8=10, Ar=90	47-20	95-96		22.9	0.058	40
17	Cr20/Al_2O_3-n	550	1.2	C_3H_8=6.67, He=93.33	33.8-17	94-96	20cycles (2 h/cycle)	2.1	0.46	41
18	10wt%Cr/MCM41	630	1.1	C_3H_8=14, N_2=86	49.7-24.9	83.8-50.0	1.67	5.2	0.65	42
19	Cr-Al-800	600	8.63	C_3H_8=100	33.2-20.4	90.4-84.7	5cycles (3 h/cycle)	25.0	0.23	43
20	Ga_2O_3/HZSM-48(Si/Al=130)	600	0.81	C_3H_8=2.5, CO_2=5, N_2=92.5	52.6-24.6	42.2-63.4	50	2.5	0.024	44
21	Ga_2O_3/HZSM-5(Si/Al=240)	600	0.81	C_3H_8=2.5, CO_2=5, N_2=92.5	57.5-48.0	40.2-45.2	30	2.6	0.013	45
22	mesoporous Ga_2O_3–m	550	6.47	C_3H_8=6.67, CO_2=33.33, He=60	19-11	91.6-95.0	4cycles (4h/cycle)	0.4	0.16	46
23	[(≡SiO)$_3$Ga(XOSi≡)]	550	10.67	C_3H_8=20,Ar=80	9.3-6.5	94.3	20	0.02 [d]	0.02	47
24	5wt%Ga_2O_3/SBA-15	620	0.6	C_3H_8=5, Ar=95	32-17	90	30	7.7	0.028	48
25	(1:1Ni:Ga)@Ni_3Ga/Al_2O_3	600	2.16	C_3H_8=10,Ar=90	13-9	94-81	82	0.4	0.005	49
26	Ga/ H-ZSM-5(Ga/Al=0.5)	510	23.6	C_3H_8=0.875,	0.5	95.1		0.4		50

				N_2=99.125						
27	Ga(i-Bu)$_3$/Al_2O_{3-500}	550	1.35	C_3H_8=20, Ar=80	24-8	90	25	23.7	0.05	51
28	0.05wt%Ru/LaZrO$_x$	550	23.6	C_3H_8=40, N_2=60	10	97		0.054[d]		52
29	0.05wt%Ru/YZrO$_x$	600	6.29	C_3H_8=40, N_2=60	48.6	85	20cycles (0.5h/cycle)	0.06 [d]		53
30	ZrO_2	550	1.57	C_3H_8=40, N_2=60	30	83	70cycles (0.5h/cycle)	0.008 [d]		54
31	Cr20Zr80/SiO_2	550	34.5	C_3H_8=40, N_2=60	30	85	50cycles (0.5h/cycle)	0.054 [d]		55
32	CuZrO-8	600	4.73	C_3H_8=13.3,Ar=86.7	25	98	10cycles (0.5h/cycle)	0.03 [d]		56
33	ZrO_2_300	550	1.44	C_3H_8=40, N_2=60	10	93	20cycles (0.5h/cycle)	0.03[d]		57
34	m-ZrO_2	550	8.63	C_3H_8=40, N_2=60	9	90		0.018 [d]		58
35	5wt%ZnO/HZSM-5(650)	600	0.27	C_3H_8=2.5, N_2=97.5	54.3-30.9	54.5-65.6	30	2.9	0.033	59
36	Zn/SiO_2	550	0.18	C_3H_8=3, Ar=97	-	95	12	0.0	0.052	60
37	2c-ZnO-Y(20.1wt%Zn)	550	400	C_3H_8=5, Ar=95	15.0-4.7	85-90	9	0.1	0.14	61
38	Pt-ZnO/Al_2O_3(0.1wt% Pt, 15wt% Zn)	600	3	C_3H_8=28, H_2=28, N_2=44	35-31	94-97	4	4.9	0.045	62
39	ALD synthesis Zn_F	550		C_3H_8=2.5, Ar=97.5	-	75	12	0.0	0.08	63

40	10wt%Zn/250HZSM-5 (SiO_2/Al_2O_3 ratio is of 250)	600	0.54	C_3H_8=5, N_2=95	74-45	62-93	10	5.5	0.12	64
41	8%ZnO-S-1_1	550	7.9	C_3H_8=6, N_2=9	33-30	87-90%	1.5	39.7	0.093	65
42	Z80Fe1.0LT	450	4.32	C_3H_8=5, N_2O=5, He=90	15-10	40-60	6.67	55.4	0.07	66
43	Fe(H)-Z1st	400	3.5	C_3H_8=1.5, N_2O=1.5, He=97	29.5-21.3	54.2-70.4	4	95.2	0.15	67
44	20Fe/5S–Al	560	0.64	C_3H_8=98.7, C_3H_6=1.3	24-11	79.1-81.8	35	0.8	0.027	68
45	10FeAl	560	0.54	C_3H_8=99	24-22	83.3-81.8	10	2.0	0.11	69
46	(3Fe:P)/Al_2O_3	600	0.27	C_3H_8=5, N_2=95	15-12	>80	5.33	0.6	0.024	70
47	Co/H-ZSM5	500	-	C_3H_8=20, He=80	-25	-60				71
48	20Co–5S/Al (20wt %Co_3O_4)	600	0.43	C_3H_8=100	50-35	70.2-71.4	6	1.4	0.15	72
49	Co/Al_2O_3(5wt%Co_3O_4)	560	0.43	C_3H_8=100	25.7-21.8	83.6-81.6	6	0.8	0.0043	73
50	Mesoporous $CoAl_2O_4$ spinel (Co-Al)(11.1wt%Co)	600	0.54	C_3H_8=5, N_2=95	39-15	75-84	5	2.0	0.26	74
51	CoAIM-Ni(II)SIM+NU-1000	230	0.126	C_3H_8=54.5,O_2=45.5	1	56	20			75
52	re-oxide Co/Al_2O_3(5.7wt%Co)	600	0.91	C_3H_8=66.67, N_2=33.33	44.7-22.5	93.0-92.1	6	8.9	0.17	76
53	Co−1.3-Zr/SiO_2	550	0.13	C_3H_8=3, Ar=97	9.5-7.3	97	20	1.1	0.014	77

54	Co/Al_2O_3-NS (Al_2O_3 nano-sheet)	550	0.98	C_3H_8=9.09, He=90.91	14.4-7.7	92.7-90.5	12	3.4	0.056	78
55	Co-Al_2O_3-HT	590	2.9	C_3H_8=20, H_2=16, N_2=64	24.8-21.1	97.1-	5	17.7	0.05	79
56	$In_2Al_8O_{15}$	600	0.15	C_3H_8=2.5, CO_2=10, N_2=87.5	35.7-29.3	76.5-77.5	20	0.01	0.058	80
57	10wt%In_2O_3/Al_2O_3	600	0.15	C_3H_8=2.5, CO_2=10, N_2=87.5	21-24-21(0.5h, 3h, 8h)	29-83-85	8	0.02	0.034	81
58	2%In_2O_3-98%Ga_2O_3	600	1.08	C_3H_8=5, N_2=95	17.5-3	32.5-55	5.83	0.0014 [d]	0.33	82
59	5%In_2O_3-15%Ga_2O_3- 80%Al_2O_3	600	1.08	C_3H_8=5, N_2=95	17.0-12.1	85.5-56.8	6	0.0046 [d]	0.067	83
60	K-CrO_x/Al_2O_3	600	1	C_3H_8=4, N_2=16	47.7-25.3	81.5-89.6	1	2.8	0.99	This work
61	VO_x/CeO_2-Al_2O_3 redox catalyst	600	1	C_3H_8=4, N_2=16	48.8-48	87.3-90.5	1	13.3	0.04	This work
62	VO_x/CeO_2-Al_2O_3 redox catalyst	600	1	C_3H_8=4, N_2=16	52.1-50.3	89.1-88.2	300cycles (0.5h/cycle)		-	This work

a. The catalysts included here are only the best-performing ones from the articles considered.
b. Formation rate is defined as mol $C_3H_6 \cdot g_M^{-1} \cdot h^{-1}$, M represents Cr, V, Ga, Zr, Zn, Fe, Co, and In in various oxide catalysts.
c. k_d, deactivation rate constant is calculated from $\ln((1-X_{final})/X_{final}) = k_d \cdot t + \ln((1-X_{initial})/X_{initial})$.
d. Formation rate is defined as mol $C_3H_6 \cdot g_{cat}^{-1} \cdot h^{-1}$

Table S5. BET and pore size changes of vanadia-ceria catalysts (6V/30CeAl) during redox cycles.

**Samples**	**BET (m^2/g)**	**Pore size (nm)**
Fresh	112.6	7.0
2 cycle	110.1	7.5
300 cycle	117.0	7.7

Table S6. Simulation Settings (modules and methods) by ASPEN plus.

**Aspen Plus modules, databanks and methods**	
Stream class	MIXCISLD
Databank	PURE, SOLIDS, INORGANIC
Property method	SRK, STEAMNBS
Unit operation models	
Reactor, regenerator, and hydrogenator	RStoic
Pressure changers	MCompr. &Valve
Heat exchangers	Heater
Distillation columns	DSTWU
Separator	Sep
Flash unit	Flash2

Table S7. Simulation conditions and assumptions by ASPEN plus.

**Ambient condition**		**T=25°C, p=1atm**
Reaction unit	Oleflex process	Reacting at 600°C, reheating at 650°C<br>Hydrogenation dilution ratio 1.4
	Chemical looping process	Reacting at 600°C<br>Regenerating excess air coeff. 1.4
Compression & refrigeration unit	Compressor	3 stages with outlet pressure at 14bar<br>Isentropic efficiency 0.72
	Intercooler	Outlet temperature at 40°C
	Cold box	Refrigeration temperature at -95°C
Separation unit	Debutanizer	80 stages at 15 bar<br>Propane recovery 0.999
	Deethanizer	70 stages at 12 bar<br>Propylene recovery 0.999
	Propylene tower	200 stages at 17 bar<br>Propylene recovery 0.995
	Pressure swing Adsorption (PSA)	Hydrogen recovery 0.98
	CO_2 scrubber	CO_2 removal efficiency 0.999
Others	Selective hydrogenator	Adiabatic with excess hydrogen coeff. 1.0
	Fuel gas combustor	Excess air coeff. 1.2<br>Exhaust gas temperature at 150°C

Table S8. Simulation Settings (feeds and yields).

	Oleflex	Chemical Looping PDH
Feed wt%	37500 kg/hr (96 propane, 2 ethane, 2 butane)	
Carbon yield mole% (propane-based)		
Propane	65.00	50.00
Propylene	31.50	43.30
Methane	0.93	2.25
Ethylene	0.93	1.13
Ethane	0.94	1.28
Allylene	0.70	0
Carbon dioxide	0	0.57
Coke(graphite)	0	1.50
Hydrogen yield mole% (propane-based)		
Hydrogen	76.96	70.20
Water	23.04	29.80

Table S9. Comparison of energy consumption of non-oxidative PDH and CL-ODH.

Section	Oleflex[60,61]		CL-ODH	
	Total MW	Unit GJ/ton C_3H_6	Total MW	Unit GJ/ton C_3H_6
**Reaction** [a]	92.13	10.75	43.95	5.36
**Compression** [b]	46.06	5.37	18.26	2.23
**Separation** [c]	254.40	29.68	155.21	18.92
**Total**	392.59	45.81	217.43	26.50
**Total**	415.28[d]			
**CO_2 emission ton/ton product**	2.51		1.51	

[a] Reaction section: reactor, regenerator, furnace (fuel gas considered), quench

[b] Compression section: cooler, compressor (intercooler included)

[c] Separation section: cold box, debutanizer, deethanizer, propylene tower, PSA, CO_2 scrubber

[d] Total energy consumption of Oleflex from the reference[62]

The total energy consumption of Oleflex in our simulation (392.59 MW) is similar to the results (415.28 MW) in reference[62], validating the reasonability of our simulations. Different forms of energy are consumed in the process, so it is helpful to convert them into the same thermal basis. Thermal to steam energy efficiency at 85% and thermal to mechanic energy efficiency at 40%. Combustion heat of the fuel gas emitted by PSA and deethanizer is calculated with the excess air coefficient at 1.2 and exhaust temperature at 150 °C. The rest heat demand is balanced by pure methane combustion, the combustion heat of which is 50.07 MJ/kg.

Table S10. Comparison of energy consumption of non-oxidative PDH and CL-ODH.

Equipment system	Oleflex		CL-PDH	
	Total MW	Unit GJ/ton C_3H_6	Total MW	Unit GJ/ton C_3H_6
**Reaction** [a]	92.13	10.75	43.95	5.36
**Compression**	46.06	5.37	18.26	2.23
**Refrigeration**	98.39	11.48	56.32	6.87
**Deethanizer**	10.30	1.20	15.79	1.93
**Debutanizer**	18.23	2.13	13.28	1.62
**Propylene tower**	80.67	9.41	56.34	6.87
**Others** [b]	46.81	5.46	13.48	1.64

[a] Reaction system contains a reactor, regenerator, and quench.

[b] Others: PSA and CO_2 scrubber.

As a result, 18.92 GJ of energy is required for separation to produce each ton of the propylene product, lower than that 29.68 GJ in Oleflex scheme. Moreover, the exothermic ODH reaction offsets the energy consumption for the reactor/regenerator, leading to a net energy consumption of 5.36 GJ/ton of propylene. Besides the energy savings for steam generation, CL-ODH is more efficient in all the other key process steps.

Table S11. The surface reaction coefficient (k_{chem}) and bulk diffusion coefficient (D_{diff}) of oxygen in vanadia (6V/Al), ceria (30CeAl) and ceria-vanadia redox catalysts (6V/30CeAl) at different temperatures were determined by analyzing the thermogravimetric relaxation kinetics.

	k_{chem} (cm/s)			D_{diff} (cm^2/s)		
**T (°C)**	**VO_x**	**CeO_2**	**VO_x-CeO_2**	**VO_x**	**CeO_2**	**VO_x-CeO_2**
**550**	-	9.23E-07	9.07E-06	-	2.51E-06	2.07E-05
**575**	-	2.23E-06	1.12E-05	-	3.66E-06	4.21E-05
**600**	-	4.00E-06	1.93E-05	-	1.14E-05	6.21E-05

Table S12. Bader charge difference for V atoms in (a) ML-V_2O_5, (b)ML-VO_2 and (c) ML-V_2O_3. The Bader charge result for pure V_2O_5 surface is taken as reference to show how electrons accumulate in ML-VO_x.

V atom number	ML-V_2O_5 (e^-)	ML-VO_2 (e^-)	ML-V_2O_3 (e^-)
1	0.02	0.01	0.02
2	-0.03	0.01	0.26
3	-0.05	0.01	0.02
4	0.00	-0.01	0.27
5	0.02	0.01	0.02
6	-0.03	-0.03	0.26
7	-0.05	0.02	0.03
8	0.00	0.00	0.24
9	0.02	0.00	0.02
10	-0.03	-0.01	0.26
11	-0.05	0.01	0.03
12	0.00	0.00	0.27
13	0.02	0.01	0.02
14	-0.03	-0.02	0.25
15	-0.05	0.01	0.03
16	0.00	-0.02	0.26
Average	-0.02	0.00	0.14

Supplementary References

1 Lei, Y. *et al.* Synthesis of Pt–Pd core–shell nanostructures by atomic layer deposition: application in propane oxidative dehydrogenation to propylene. *Chem. Mater.* **24**, 3525-3533 (2012).

2 Sugiyama, S. *et al.* Application of the unique redox properties of magnesium ortho-vanadate incorporated with palladium in the unsteady-state operation of the oxidative dehydrogenation of propane. *J. Catal.* **260**, 157-163 (2008).

3 Shi, L. *et al.* Edge-hydroxylated boron nitride for oxidative dehydrogenation of propane to propylene. *ChemCatChem* **9**, 1788-1793 (2017).

4 Grant, J. T. *et al.* Selective oxidative dehydrogenation of propane to propene using boron nitride catalysts. *Science* **354**, 1570-1573 (2016).

5 Zhang, X., Yue, Y. & Gao, Z. Chromium oxide supported on mesoporous SBA-15 as propane dehydrogenation and oxidative dehydrogenation catalysts. *Catal. Lett.* **83**, 19-25 (2002).

6 Abello, M. C., Gomez, M. F. & Ferretti, O. Mo/γ-Al_2O_3 catalysts for the oxidative dehydrogenation of propane. Effect of Mo loading. *Appl. Catal. A* **207**, 421-431 (2001).

7 Mitran, G. *et al.* Propane oxidative dehydrogenation over VO_x/SBA-15 catalysts. *Catal. Today* **306**, 260-267 (2018).

8 Schwarz, O. *et al.* Impact of preparation method on physico-chemical and catalytic properties of VO_x/γ-Al_2O_3 materials. *J. Mol. Catal. A* **293**, 45-52 (2008).

9 Carrero, C. *et al.* High performance (VO_x)n–(TiO_x)m/SBA-15 catalysts for the oxidative dehydrogenation of propane. *Catal. Sci. Technol.* **4**, 786 (2014).

10 Lemonidou, A. A., Nalbandian, L. & Vasalos, I. A. Oxidative dehydrogenation of propane over vanadium oxide based catalysts Effect of support and alkali promoter. *Catal. Today* **61**, 333–341 (2000).

11 Fattahi, M., Kazemeini, M., Khorasheh, F. & Rashidi, A. Kinetic modeling of oxidative dehydrogenation of propane (ODHP) over a vanadium–graphene catalyst: Application of the DOE and ANN methodologies. *J. Ind. Eng. Chem.* **20**, 2236-2247 (2014).

12 Yan, H. *et al.* Tandem In_2O_3-Pt/Al_2O_3 catalyst for coupling of propane dehydrogenation to selective H_2 combustion. *Science* **371**, 1257–1260 (2021).

13 Michorczyk, P., Pietrzyk, P. & Ogonowski, J. Preparation and characterization of SBA-1–supported chromium oxide catalysts for CO_2 assisted dehydrogenation of propane. *Micro. Mesop. Mater.* **161**, 56-66 (2012).

14 Gao, X.-Q., Lu, W.-D., Hu, S.-Z., Li, W.-C. & Lu, A.-H. Rod-shaped porous alumina-supported Cr2O3 catalyst with low acidity for propane dehydrogenation. *Chinese J. Catal.* **40**, 184-191 (2019).

15 Otroshchenko, T. P., Rodemerck, U., Linke, D. & Kondratenko, E. V. Synergy effect between Zr and Cr active sites in binary $CrZrO_x$ or supported $CrO_x/LaZrO_x$: Consequences for catalyst activity, selectivity and durability in non-oxidative propane dehydrogenation. *J. Catal.* **356**, 197-205 (2017).

16 Zhao, Z.-J. *et al.* Hydroxyl-Mediated Non-oxidative Propane Dehydrogenation over VO_x/γ-Al_2O_3 Catalysts with Improved Stability. *Angew. Chem. Int. Ed.* **57**, 6791-6795 (2018).

17 Chen, C. *et al.* Nature of active phase of VO_x catalysts supported on SiBeta for direct dehydrogenation of propane to propylene. *Chinese J. Catal.* **41**, 276-285 (2020).

18 Sokolov, S., Stoyanova, M., Rodemerck, U., Linke, D. & Kondratenko, E. V. Comparative study of propane dehydrogenation over V-, Cr-, and Pt-based catalysts: Time on-stream behavior and origins of deactivation. *J. Catal.* **293**, 67-75 (2012).

19 Sokolov, S., Stoyanova, M., Rodemerck, U., Linke, D. & Kondratenko, E. V. Effect of support on selectivity and on-stream stability of surface VO_x species in non-oxidative propane dehydrogenation. *Catal. Sci. Technol.* **4**, 1323-1332 (2014).

20 Sokolov, S. *et al.* Effect of VO_x Species and support on coke formation and catalyst stability in nonoxidative propane dehydrogenation. *ChemCatChem* **7**, 1691-1700 (2015).

21 Wu, T. *et al.* Structure and catalytic consequence of Mg-modified VO_x/Al_2O_3 catalysts

for propane dehydrogenation. *AIChE J.* **63**, 4911-4919 (2017).
22 Liu, G., Zhao, Z.-J., Wu, T., Zeng, L. & Gong, J. Nature of the active sites of VO_x/Al_2O_3 catalysts for propane dehydrogenation. *ACS Catal.* **6**, 5207-5214 (2016).
23 Zhu, H. *et al.* Sn surface-enriched Pt–Sn bimetallic nanoparticles as a selective and stable catalyst for propane dehydrogenation. *J. Catal.* **320**, 52-62 (2014).
24 Li, B., Xu, Z., Jing, F., Luo, S. & Chu, W. Facile one-pot synthesized ordered mesoporous Mg-SBA-15 supported PtSn catalysts for propane dehydrogenation. *Appl. Catal. A* **533**, 17-27 (2017).
25 Jang, E. J., Lee, J., Jeong, H. Y. & Kwak, J. H. Controlling the acid-base properties of alumina for stable PtSn-based propane dehydrogenation catalysts. *Appl. Catal. A* **572**, 1-8 (2019).
26 Xu, Z., Yue, Y., Bao, X., Xie, Z. & Zhu, H. Propane dehydrogenation over Pt clusters localized at the Sn single-site in zeolite framework. *ACS Catal.* **10**, 818-828 (2019).
27 Grasselli, R. K., Stern, D. L. & Tsikoyiannis, J. G. Catalytic dehydrogenation (DH) of light paraffins combined with selective hydrogen combustion (SHC) I. DH→SHC→DH catalysts in series (co-fed process mode). *Appl. Catal. A* **189**, 1-8 (1999).
28 Chen, S. *et al.* Modulating lattice oxygen in dual-functional Mo-V-O mixed oxides for chemical looping oxidative dehydrogenation. *J. Am. Chem. Soc.* **141**, 18653-18657 (2019).
29 Crapanzano, S., Babich, I. V. & Lefferts, L. Selection of mixed conducting oxides for oxidative dehydrogenation of propane with pulse experiments. *Appl. Catal. A* **391**, 70-77 (2011).
30 Hossain, M. M. Kinetics of oxidative dehydrogenation of propane to propylene using lattice oxygen of $VO_x/CaO/\gamma Al_2O_3$ catalysts. *Ind. Eng. Chem. Res.* **56**, 4309-4318 (2017).
31 Bai, P. *et al.* Relationship between surface chemistry and catalytic performance of mesoporous γ-Al_2O_3 supported VO_X catalyst in catalytic dehydrogenation of propane. *ACS Appl. Mater. Int.* **8**, 25979-25990 (2016).
32 Liu, G., Zhao, Z.-J., Wu, T., Zeng, L. & Gong, J. Nature of the Active Sites of VO_x/Al_2O_3 Catalysts for Propane Dehydrogenation. *ACS Catal.* **6**, 5207-5214 (2016).
33 Rodemerck, U., Stoyanova, M., Kondratenko, E. V. & Linke, D. Influence of the kind of VO_x structures in VO_x/MCM-41 on activity, selectivity and stability in dehydrogenation of propane and isobutane. *J. Catal.* **352**, 256-263 (2017).
34 Hu, P., Lang, W.-Z., Yan, X., Chu, L.-F. & Guo, Y.-J. Influence of gelation and calcination temperature on the structure-performance of porous VO_X-SiO_2 solids in non-oxidative propane dehydrogenation. *J. Catal.* **358**, 108-117 (2018).
35 Hu, P., Lang, W.-Z., Yan, X., Chen, X.-F. & Guo, Y.-J. Vanadium-doped porous silica materials with high catalytic activity and stability for propane dehydrogenation reaction. *Appl. Catal. A* **553**, 65-73 (2018).
36 Yang, Q.-Q., Hu, P., Xiu, N.-Y., Lang, W.-Z. & Guo, Y.-J. VO_x/γ-Al_2O_3 Catalysts for propane dehydrogenation prepared by "impregnation-solid phase reaction" method with aluminum hydroxide as support precursor. *Chem. Select* **3**, 10049-10055 (2018).
37 Shee, D. & Sayari, A. Light alkane dehydrogenation over mesoporous Cr_2O_3/Al_2O_3 catalysts. *Appl. Catal. A* **389**, 155-164 (2010).
38 Węgrzyniak, A. *et al.* Catalytic behaviour of chromium oxide supported on CMK-3 carbon replica in the dehydrogenation propane to propene. *Appl. Catal. A* **508**, 1-9 (2015).
39 Lang, W.-Z., Hu, C.-L., Chu, L.-F. & Guo, Y.-J. Hydrothermally prepared chromia-alumina (xCr/Al_2O_3) catalysts with hierarchical structure for propane dehydrogenation. *RSC Adv.* **4**, 37107-37113 (2014).
40 Li, P.-P. *et al.* The promotion effects of Ni on the properties of Cr/Al catalysts for propane dehydrogenation reaction. *Appl. Catal. A* **522**, 172-179 (2016).
41 Węgrzyniak, A. *et al.* Catalytic behavior of chromium oxide supported on nanocasting-prepared mesoporous alumina in dehydrogenation of propane. *Nanomaterials* **7** (2017).
42 He, D., Zhang, Y., Yang, S., Mei, Y. & Luo, Y. Investigation of the isolated Cr(VI) species in Cr/MCM-41 catalysts and its effect on catalytic activity for dehydrogenation of propane. *ChemCatChem* **10**, 5434-5440 (2018).

43 Gao, X.-Q., Lu, W.-D., Hu, S.-Z., Li, W.-C. & Lu, A.-H. Rod-shaped porous alumina-supported Cr_2O_3 catalyst with low acidity for propane dehydrogenation. *Chinese J. Catal.* **40**, 184-191 (2019).
44 Ren, Y., Wang, J., Hua, W., Yue, Y. & Gao, Z. Ga_2O_3/HZSM-48 for dehydrogenation of propane: Effect of acidity and pore geometry of support. *J. Ind. Eng. Chem.* **18**, 731-736 (2012).
45 Barnes, D. G. *et al.* Embedding and publishing interactive, 3-dimensional, scientific figures in Portable Document Format (PDF) files. *PLoS One* **8**, e69446 (2013).
46 Michorczyk, P., Kuśtrowski, P., Kolak, A. & Zimowska, M. Ordered mesoporous Ga_2O_3 and Ga_2O_3–Al_2O_3 prepared by nanocasting as effective catalysts for propane dehydrogenation in the presence of CO_2. *Catal. Commun.* **35**, 95-100 (2013).
47 Searles, K., Siddiqi, G., Safonova, O. V. & Coperet, C. Silica-supported isolated gallium sites as highly active, selective and stable propane dehydrogenation catalysts. *Chem Sci* **8**, 2661-2666 (2017).
48 Shao, C.-T., Lang, W.-Z., Yan, X. & Guo, Y.-J. Catalytic performance of gallium oxide based-catalysts for the propane dehydrogenation reaction: effects of support and loading amount. *RSC Adv.* **7**, 4710-4723 (2017).
49 He, Y., Song, Y., Cullen, D. A. & Laursen, S. Selective and stable non-noble-metal intermetallic compound catalyst for the direct dehydrogenation of propane to propylene. *J. Am. Chem. Soc.* **140**, 14010-14014 (2018).
50 Schreiber, M. W. *et al.* Lewis-bronsted acid pairs in Ga/H-ZSM-5 to catalyze dehydrogenation of light alkanes. *J. Am. Chem. Soc.* **140**, 4849-4859 (2018).
51 Szeto, K. C. *et al.* A Strong support effect in selective propane dehydrogenation catalyzed by $Ga(i\text{-}Bu)_3$ grafted onto γ-alumina and silica. *ACS Catal.* **8**, 7566-7577 (2018).
52 Otroshchenko, T. *et al.* ZrO_2-based alternatives to conventional propane dehydrogenation catalysts: active sites, design, and performance. *Angew. Chem. Int. Ed. Engl.* **54**, 15880-15883 (2015).
53 Otroshchenko, T., Kondratenko, V. A., Rodemerck, U., Linke, D. & Kondratenko, E. V. ZrO_2 -based unconventional catalysts for non-oxidative propane dehydrogenation: Factors determining catalytic activity. *J. Catal.* **348**, 282-290 (2017).
54 Zhang, Y. *et al.* Control of coordinatively unsaturated Zr sites in ZrO_2 for efficient C-H bond activation. *Nat. Commun.* **9**, 3794 (2018).
55 Han, S. *et al.* Unravelling the origins of the synergy effect between ZrO_2 and CrO_x in supported $CrZrO_x$ for propene formation in non-oxidative propane dehydrogenation. *ACS Catal.* 2020, 10, 2, 1575–1590 (2019).
56 Jeon, N., Choe, H., Jeong, B. & Yun, Y. Cu-promoted zirconia catalysts for non-oxidative propane dehydrogenation. *Appl. Catal. A* **586** (2019).
57 Otroshchenko, T. *et al.* Controlling activity and selectivity of bare ZrO_2 in non-oxidative propane dehydrogenation. *Appl. Catal. A* **585** (2019).
58 Zhang, Y. *et al.* The effect of phase composition and crystallite size on activity and selectivity of ZrO_2 in non-oxidative propane dehydrogenation. *J. Catal.* **371**, 313-324 (2019).
59 Zhang, F., Miao, C., Yue, Y., Hua, W. & Gao, Z. Dehydrogenation of propane to propylene in the presence of CO_2 over steaming-treated hzsm-5 supported ZnO. *Chinese J. Chem.* **30**, 929-934 (2012).
60 Schweitzer, N. M. *et al.* Propylene hydrogenation and propane dehydrogenation by a single-site Zn^{2+} on silica catalyst. *ACS Catal.* **4**, 1091-1098 (2014).
61 Gong, T., Qin, L., Lu, J. & Feng, H. ZnO modified ZSM-5 and Y zeolites fabricated by atomic layer deposition for propane conversion. *Phys. Chem. Chem. Phys.* **18**, 601-614 (2016).
62 Liu, G. *et al.* Platinum-modified ZnO/Al_2O_3 for propane dehydrogenation: minimized platinum usage and improved catalytic stability. *ACS Catal.* **6**, 2158-2162 (2016).
63 Camacho-Bunquin, J. *et al.* Single-site zinc on silica catalysts for propylene hydrogenation and propane dehydrogenation: Synthesis and reactivity evaluation using an integrated atomic layer deposition-catalysis instrument. *J. Catal.* **345**, 170-182 (2017).
64 Chen, C. *et al.* ZnO supported on high-silica HZSM-5 as efficient catalysts for direct

dehydrogenation of propane to propylene. *Mol. Catal.* **476** (2019).
65 Zhao, D. *et al.* In situ formation of ZnO_x species for efficient propane dehydrogenation. *Nature* **599**, 234-238 (2021).
66 Ates, A., Hardacre, C. & Goguet, A. Oxidative dehydrogenation of propane with N_2O over Fe-ZSM-5 and Fe–SiO_2: Influence of the iron species and acid sites. *Appl. Catal. A* **441-442**, 30-41 (2012).
67 Sazama, P. *et al.* Structure and critical function of Fe and acid sites in Fe-ZSM-5 in propane oxidative dehydrogenation with N_2O and N_2O decomposition. *J. Catal.* **299**, 188-203 (2013).
68 Sun, Y.-n., Tao, L., You, T., Li, C. & Shan, H. Effect of sulfation on the performance of Fe_2O_3/Al_2O_3 catalyst in catalytic dehydrogenation of propane to propylene. *Chem. Eng. J.* **244**, 145-151 (2014).
69 Sun, Y. *et al.* Effect of pre-reduction on the performance of Fe_2O_3/Al_2O_3 catalysts in dehydrogenation of propane. *J. Mol. Catal. A* **397**, 120-126 (2015).
70 Tan, S. *et al.* Propane dehydrogenation over alumina-supported iron/phosphorus catalysts: structural evolution of iron species leading to high activity and propylene selectivity. *ACS Catal.* **6**, 5673-5683 (2016).
71 Yu, S. Y., Yu, G. J., Li, W. & Iglesia, E. Kinetics and reaction pathways for propane dehydrogenation and aromatization on Co/H-ZSM5 and H-ZSM5. *J. Phys. Chem. B* 4714-4720 (2002).
72 Sun, Y.-n. *et al.* Effect of sulfate addition on the performance of Co/Al_2O_3 catalysts in catalytic dehydrogenation of propane. *Catal. Commun.* **60**, 42-45 (2015).
73 Sun, Y., Wu, Y., Shan, H. & Li, C. Studies on the nature of active cobalt species for the production of methane and propylene in catalytic dehydrogenation of propane. *Catal. Lett.***145**, 1413-1419 (2015).
74 Hu, B. *et al.* A Mesoporous cobalt aluminate spinel catalyst for nonoxidative propane dehydrogenation. *ChemCatChem* **9**, 3330-3337 (2017).
75 Li, Z. *et al.* Fine-tuning the activity of metal-organic framework-supported cobalt catalysts for the oxidative dehydrogenation of propane. *J. Am. Chem. Soc.* **139**, 15251-15258 (2017).
76 Li, X., Wang, P., Wang, H. & Li, C. Effects of the state of Co species in Co/Al_2O_3 catalysts on the catalytic performance of propane dehydrogenation. *Appl. Sur. Sci.* **441**, 688-693 (2018).
77 Zhao, Y. *et al.* Zirconium modification promotes catalytic activity of a single-site cobalt heterogeneous catalyst for propane dehydrogenation. *ACS Omega* **3**, 11117-11127 (2018).
78 Dewangan, N. *et al.* Cobalt-based catalyst supported on different morphologies of alumina for non-oxidative propane dehydrogenation: effect of metal support interaction and lewis acidic sites. *ChemCatChem* **11**, 4923-4934 (2019).
79 Dai, Y. *et al.* γ-Al_2O_3 sheet-stabilized isolate Co^{2+} for catalytic propane dehydrogenation. *J. Catal.* **381**, 482-492 (2020).
80 Chen, M. *et al.* Supported indium oxide as novel efficient catalysts for dehydrogenation of propane with carbon dioxide. *Appl. Catal. A* **377**, 35-41 (2010).
81 Chen, M. *et al.* Study in support effect of In_2O_3/MO_x (M=Al, Si, Zr) catalysts for dehydrogenation of propane in the presence of CO_2. *Appl. Catal. A* **407**, 20-28 (2011).
82 Tan, S. *et al.* Catalytic propane dehydrogenation over In_2O_3–Ga_2O_3 mixed oxides. *Appl. Catal. A* **498**, 167-175 (2015).
83 Tan, S. *et al.* Propane dehydrogenation over In_2O_3-Ga_2O_3-Al_2O_3 mixed oxides. *ChemCatChem* **8**, 214-221 (2016).
